# RAB5 is a host dependency factor for the generation of SARS-CoV-2 replication organelles

**DOI:** 10.1128/mbio.03314-24

**Published:** 2025-04-01

**Authors:** Yuexuan Chen, Susanne Klute, Konstantin Maria Johannes Sparrer, Ruth Serra-Moreno

**Affiliations:** 1 Microbiology and Immunology, University of Rochester Medical Center 6923, Rochester, New York, USA; 2 Institute of Molecular Virology, Ulm University Medical Center 27197, Ulm, Germany; 3 German Center for Neurodegenerative Diseases (DZNE), Ulm, Germany; Columbia University Medical Center, New York, New York, USA; University of Wisconsin-Madison, Madison, Wisconsin, USA

**Keywords:** COP-I, COPB1, NSP6, RAB5, replication organelle, SARS-CoV-2

## Abstract

Severe acute respiratory syndrome coronavirus 2 (SARS-CoV-2) remains a threat due to the emergence of variants with increased transmissibility and enhanced escape from immune responses. Like other coronaviruses before, SARS-CoV-2 likely emerged after its transmission from bats. The successful propagation of SARS-CoV-2 in humans might have been facilitated by usurping evolutionarily conserved cellular factors to execute crucial steps in its life cycle, such as the generation of replication organelles—membrane structures where coronaviruses assemble their replication-transcription complex. In this study, we found that RAB5, which is highly conserved across mammals, is a critical host dependency factor for the replication of the SARS-CoV-2 genome. Our results also suggest that SARS-CoV-2 uses RAB5^+^ membranes to build replication organelles with the aid of COPB1, a component of the COP-I complex, and that the virus protein NSP6 participates in this process. Hence, targeting NSP6 represents a promising approach to interfere with SARS-CoV-2 RNA synthesis and halt its propagation.

**IMPORTANCE:**

In this study, we sought to identify the host dependency factors that severe acute respiratory syndrome coronavirus 2 (SARS-CoV-2) uses for the generation of replication organelles: cellular membranous structures that SARS-CoV-2 builds in order to support the replication and transcription of its genome. We uncovered that RAB5 is an important dependency factor for SARS-CoV-2 replication and the generation of replication organelles, and that the viral protein NSP6 participates in this process. Hence, NSP6 represents a promising target to halt SARS-CoV-2 replication.

## INTRODUCTION

The coronavirus disease 2019 (COVID-19) pandemic has evidenced that spillover episodes of viruses naturally infecting other animals are more frequent than in previous decades (1). Besides severe acute respiratory syndrome coronavirus 2 (SARS-CoV-2), six other human coronaviruses [NL63, 229E, OC43, HKU1, SARS-CoV, and Middle East respiratory syndrome-related coronavirus (MERS-CoV)] emerged in the 20th and 21st centuries, most likely because of cross-species transmission events from bats and rodents (2–4). Human activities like deforestation, intensive livestock production, and urbanization of the environment are drivers for the increased incidence of zoonotic infections (5, 6). While higher exposure to wild animals rises the probability of transmission of their viruses, these viruses must find a suitable environment in the new host to successfully cross the species barrier. This requires (i) an appropriate receptor for entry into the new host cells, (ii) ability to antagonize/evade the antiviral defenses in the new host, and (iii) being capable of hijacking the cellular machinery (dependency factors) of the new host in order to replicate (6–8). Here, we investigated the human dependency factors that SARS-CoV-2 utilizes to generate replication organelles (ROs), a critical step in the replication cycle of coronaviruses.

Upon entry, coronaviruses synthesize the non-structural proteins (NSPs) that are responsible for the replication of their genome and production of subgenomic mRNAs, which allow the synthesis of their structural and accessory proteins. Among this first set of proteins, coronaviruses use NSP3, NSP4, and NSP6 to remodel cellular membranes and create ROs (9–13): double membrane vesicles (DMVs) where the virus replication-transcription complex (RTC) assembles (14). Most of the work on SARS-CoV-2 ROs has focused on understanding the virus molecules involved in DMV biogenesis. These studies found that while NSP3/4/6 are required to build ROs in most coronaviruses (9, 11–13), NSP3 and NSP4 are sufficient to generate DMVs in SARS-CoV-2, and that NSP6 participation is limited to increasing their number and organization, so they become functional replication factories (10). This role of NSP6 was confirmed using K22, a pan-NSP6 inhibitor that significantly reduced the number of DMVs and caused a defect in SARS-CoV-2 RNA yields (10). K22 was previously discovered in a screen for compounds against the human coronavirus 229E. Since only viruses harboring mutations in NSP6 replicated in the presence of this drug, it was concluded that K22 targets NSP6, and this was later confirmed for other coronaviruses (15). Of note, SARS-CoV-2 NSP6 also facilitates immune evasion (16, 17). However, it is unknown whether K22 impacts this activity.

Despite our increasing understanding of the virus genes involved in RO formation, the cellular origin of these replication factories is still a focus of debate. Endoplasmic reticulum (ER), Golgi, and autophagosomes have been proposed as membrane sources to generate SARS-CoV-2 ROs (11, 18–22). Structural studies revealed that these DMVs resemble autophagic membranes, suggesting that coronaviruses utilize elements of the autophagy machinery for RO biogenesis (23–29). However, autophagy has been found dispensable for the assembly of the SARS-CoV-2 RTC (10, 30). More recent studies have shown a physical connection between the SARS-CoV-2 ROs and zippered ER membranes, suggesting that ROs have an ER origin (10, 14, 31–33). Still, the dependency factors used by SARS-CoV-2 to create ROs, and whether ER membranes are their only source, remain largely unknown. Consistent with previous studies, here, we show that functional autophagy is dispensable for SARS-CoV-2 genome replication, and we identify RAB5 as a critical host dependency factor for SARS-CoV-2 ROs. RAB5 (Ras-associated protein RAB5A) is a GTPase crucial for the early steps of the endocytic pathway by facilitating vesicular transport, membrane trafficking, and signaling (34–37). Besides RAB5, our results indicate that SARS-CoV-2 builds ROs with the aid of COPB1 (COP-I coat complex subunit beta 1), a molecule of the COP-I complex, which participates in the retrograde transport from Golgi to the ER (38, 39). Finally, our studies suggest that the virus protein NSP6 recruits RAB5 and COPB1 for RO biogenesis. These conclusions have been derived from five main findings: (i) deletion of *RAB5* and *COPB1* significantly reduces SARS-CoV-2 RNA synthesis, (ii) *RAB5* deletion significantly impairs RO formation, (iii) NSP6 and the virus RNA-dependent RNA polymerase co-purify with the RAB5^+^ endosomal fraction, (iv) NSP6 associates with both RAB5 and COPB1, and (v) NSP6 facilitates an interaction between RAB5 and COPB1. Therefore, our findings reveal NSP6 as a promising target against SARS-CoV-2 and identify the endosomal and trafficking machinery as key contributors to the formation of ROs. Importantly, RAB5 and COPB1 are highly conserved in mammals (40). Hence, the use of such ubiquitous pathways might have contributed to the successful transmission of SARS-CoV-2 from its reservoir to humans.

## RESULTS

### SARS-CoV-2 RNA kinetics

**Fig 1:**
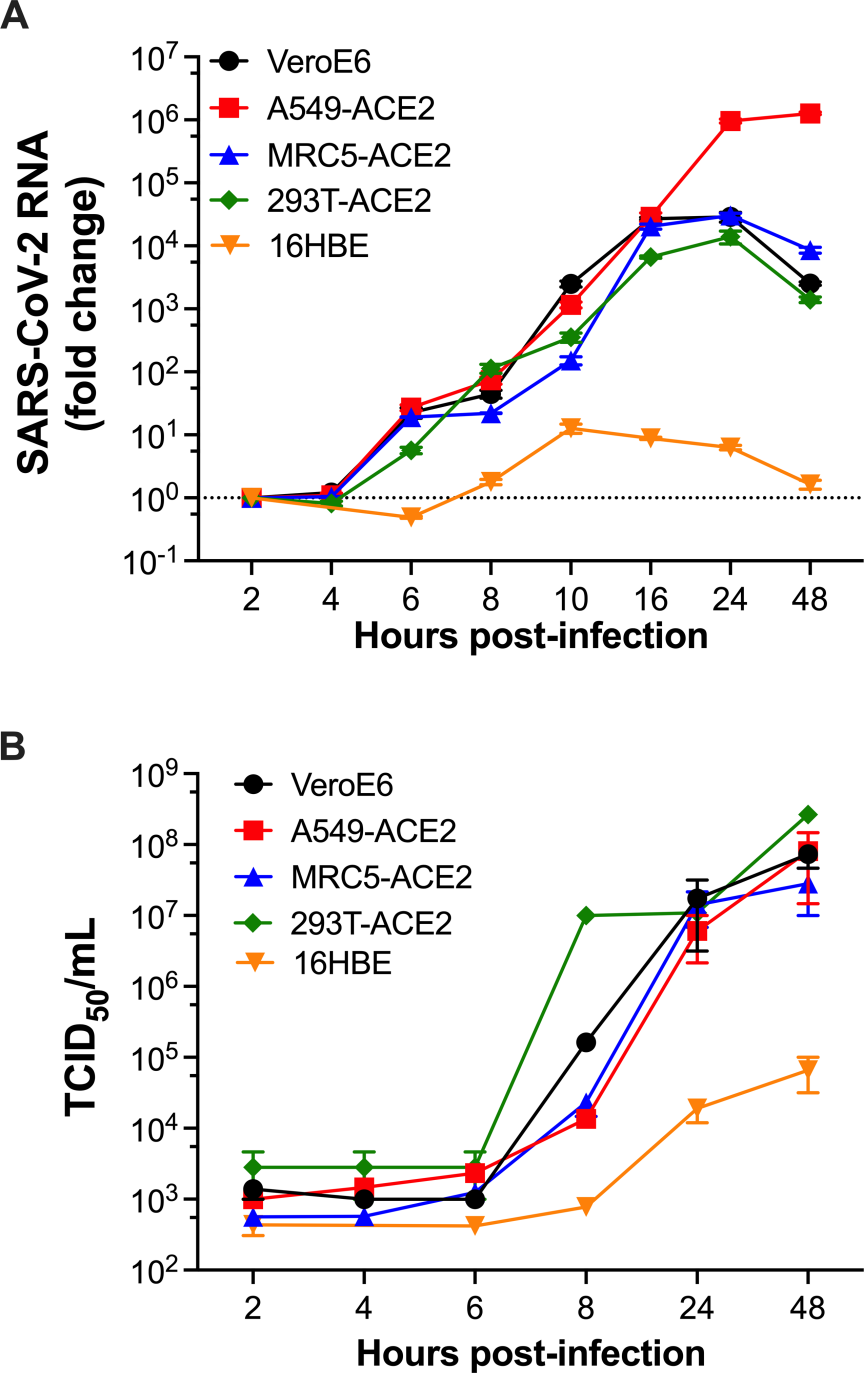
RNA replication kinetics of SARS-CoV-2. (A) VeroE6, A549-ACE2, MRC5-ACE2, HEK293T-ACE2, and 16HBE cells were synchronously infected with SARS-CoV-2 HK at MOI = 1. Total RNA was extracted at the selected time points, converted to cDNA by reverse transcription, and virus genome copies were measured by qPCR using primer pairs specific for *NSP12* and *NSP6*. Changes in SARS-CoV-2 RNA levels were normalized to the first time point and expressed as fold-change. Dotted line represents basal virus RNA counts. (B) The culture supernatants from these infections were collected at the selected time points to measure the degree of virion production by TCID_50_ on VeroE6 cells. Data correspond to the mean and SEM of three independent experiments.

To investigate the origins of the DMVs that SARS-CoV-2 uses for the assembly of the RTC, we first defined the time post-infection in which new virus RNA copies are synthesized, before progeny virions are detected. At this time, the virus-mediated remodeling of cellular membranes should have already materialized, with most products of genome replication [i.e., double-stranded RNA (dsRNA)] present in ROs (41–43). For this, we examined the RNA replication kinetics of an early SARS-CoV-2 isolate, Hong Kong (HK; GenBank: MT547814) in different cell lines. African green monkey kidney epithelial VeroE6, human lung epithelial A549-ACE2, human lung fibroblast MRC5-ACE2, human kidney HEK293T-ACE2, and human bronchial epithelial 16HBE cells were infected with SARS-CoV-2 HK at a multiplicity of infection (MOI) of 1 in a synchronized manner by keeping infections for 1 h at 4°C. At 2, 4, 6, 8, 10, 16, 24, and 48 h post-infection, cells were harvested for RNA extraction. In parallel, culture supernatants were collected to measure virion production by tissue culture infectious dose 50 (TCID_50_). SARS-CoV-2 genomic RNA was measured by reverse transcription followed by quantitative PCR (RT-qPCR) using primers specific for NSP12 (the catalytic subunit of the virus RNA-dependent RNA polymerase; RdRp) and NSP6, which both are part of *ORF1ab*, a reading frame only present in the full-length genomic RNA. qPCR primers for the host genes *GAPDH*, *ACTB,* and *CRT* were used for normalization and to ensure comparable input RNA across samples. SARS-CoV-2 genome replication was calculated as fold-change over basal virus RNA (detected at 2 h post-infection). New virus RNA copies were detected at 6 h post-infection for most cell lines—which aligns with the emergence of ROs (44, 45)—except for 16HBE cells. In this case, RNA replication was detected at 10 h post-infection (Fig. 1A). Hence, these findings indicate that the assembly of the virus RTC has already occurred by 6 h post-infection (10 h in 16HBE cells). This is consistent with an absence of infectious particle production at that time. In fact, cell-free infectious particles were first detected 8 h post-infection for most cells and at 24 h post-infection for 16HBE cells (Fig. 1B). Therefore, we selected 6 h post-infection as the time to examine the subcellular distribution of the RTC and to measure any defects in virus RNA synthesis in the presence of inhibitors for different cellular processes. For 16HBE cells, these assays were performed 10 h post-infection.

### Autophagy is dispensable for SARS-CoV-2 RNA synthesis

**Fig 2:**
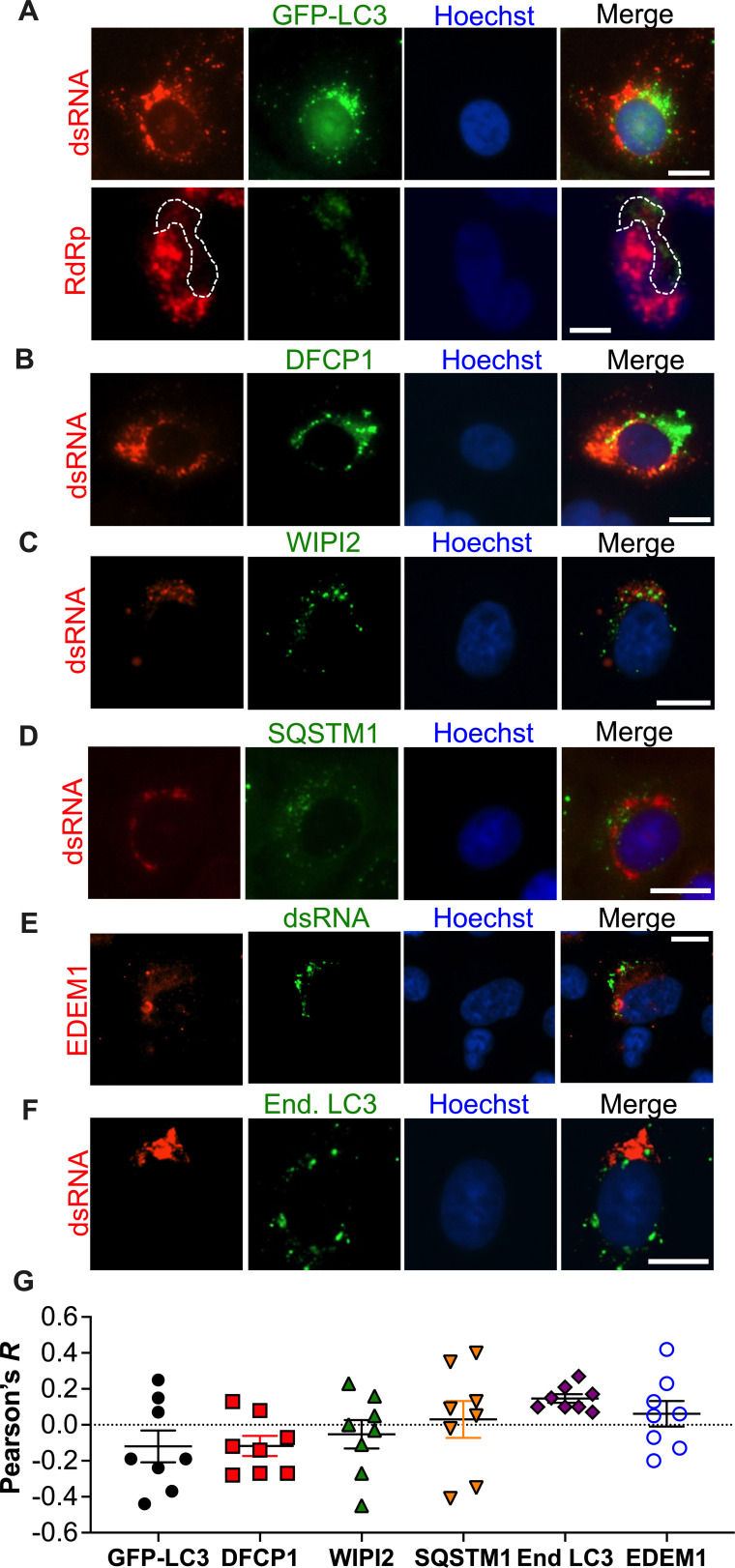
Autophagy membranes do not overlap with the SARS-CoV-2 RTC. (A–F) VeroE6 cells were infected with SARS-CoV-2 HK (MOI = 1) in a synchronized manner. Six hours later, cells were stained for the virus dsRNA and/or RdRp and different autophagy markers. Images: representative pictures from three independent experiments for the relative distribution between SARS-CoV-2 dsRNA and/or RdRp and autophagosomes (EGFP-LC3B), phagophores (DFCP1), omegasomes (WIPI2), autophagy receptors (SQSTM1), EDEMosomes (EDEM1), and endogenous LC3. Dotted white lines: delineation of cellular cytoplasm. White scale bar: 10 µm. (G) Graph: the Pearson’s correlation coefficient (*R*) value for the co-localization of SARS-CoV-2 dsRNA with the indicated markers was calculated from eight randomly selected fields. Data correspond to raw values, their mean, and SEM.

To investigate whether SARS-CoV-2 uses autophagy to generate ROs, Vero E6 cells were infected with SARS-CoV-2 HK (MOI = 1), and the distribution of the RTC relative to autophagy membrane markers was examined by fluorescence microscopy at 6 h post-synchronized infection. SARS-CoV-2 dsRNA (as an intermediate product generated during genome replication) and the virus RdRp (NSP12) were used as markers for the RTC. Specificity for these markers was tested by staining uninfected cells. While no signal for dsRNA was detected in uninfected cells, cross-reactivity of the antibody used for the virus RdRp with nuclear factors was observed (Fig. S1). However, in infected cells, RdRp staining was also found in the cytoplasm (Fig. 2A, white, dotted area), which is the location where the virus polymerase is expected (46–48). Given the RdRp unspecific nuclear staining, we subsequently decided to label the RTC with dsRNA. Our assays showed little or no overlap between the RTC and EGFP-LC3B (Fig. 2A), an autophagosome marker (49), and this was confirmed by measuring the Pearson’s correlation coefficient (*R*) (Fig. 2G). Similarly, little or no co-localization was observed between the virus RTC and phagophore (DFCP1) or omegasome (WIPI2) membranes markers (50, 51). Likewise, the autophagy receptor SQSTM1 (52, 53) distributed away from the virus RTC (Fig. 2B through D and G). Since previous work with the betacoronavirus murine hepatitis virus (MHV) uncovered that its RTC assembles in EDEMosomes (29), ER-derived vesicles that are coated with non-lipidated LC3, we investigated if SARS-CoV-2 uses a similar mechanism. However, no co-localization was detected between the RTC and the EDEMosome marker EDEM1 or endogenous LC3 (Fig. 2E through G). Here, we stained for the endogenous protein because that same study reported that EGFP-LC3B does not recapitulate the distribution of non-lipidated LC3 when coating EDEMosomes (29). Consistent with our findings in Vero E6 cells, no overlap between the virus RTC and endogenous LC3 was observed either in more physiologically relevant cells, such as A549-ACE2, MRC5-ACE2, and HEK293T-ACE2 (Fig. S2). Hence, these findings indicate that the SARS-CoV-2 RTC does not localize to autophagy-associated membranes.

To confirm that autophagy is dispensable for RO formation, we blocked autophagy by generating *ATG5* knockout cells in VeroE6, A549-ACE2, MRC5-ACE2, and HEK293T-ACE2 cells. *ATG5* was targeted because it is critical for autophagy progression and autophagosome biogenesis (54, 55). *ATG5* knockout was verified by western blot from pooled cells, showing undetectable levels of the protein and a defect in the emergence of LC3-II (Fig. S3A)—a hallmark of a defect in *ATG5* (56, 57). The effect of *ATG5* knockout on SARS-CoV-2 RNA counts was investigated by RT-qPCR at 2 and 6 h post-infection to assess whether *ATG5* deletion causes any defects in genome replication due to impacting virus uptake. To facilitate data interpretation, RT-qPCR data are expressed as the inverse value of the raw Cq (1/Cq), with no normalization, but RT-qPCR for cellular *GAPDH*, *ACTB,* and *CRT* confirmed comparable input RNA across samples. The reasoning for presenting the data in this format is that the Cq values for the virus RNA earlier in the infection are higher than when genome replication is occurring. So, plotting the raw Cq would have given the impression that there is a decrease in RNA levels over time. Through this method, we can see the magnitude of the impact (if any) that knocking out *ATG5* has on genome replication. Nevertheless, because this is an unusual presentation of qPCR data, this information is also provided as fold-change over parental cells using the 2^ΔΔCq^ factor (Fig. S4A). Deletion of *ATG5* had no negative impact on SARS-CoV-2 RNA uptake, since the Cq values were comparable between the respective parental and *ATG5*KO cells at 2 h post-infection (Fig. S3A, open circles). Similarly, *ATG5* deletion had no effect on virus RNA synthesis in any of the cell types investigated at 6 h post-infection or at later time points (Fig. S3A and S4A). In *ATG5*KO A549-ACE2 cells, however, RNA yields were reduced at 24 h post-infection. Since no defect in RNA synthesis was observed in these cells at 6 h post-infection, we concluded that autophagy is important for late steps of the virus life cycle in this cell line, like virion assembly and/or egress. This is consistent with reduced virus RNA counts 24 h post-infection, which would correspond to ~3 replication cycles.

To further rule out a role for autophagy in RO biogenesis, we used the following autophagy-modulating compounds: 3-MA (class III PI3K inhibitor with transient activity on class I PI3K), VPS34IN (class III PI3K inhibitor), torin2, vistusertib (mTOR inhibitors), dactolisib (PI3K [p110α/γ/δ/β] and mTOR inhibitor), and wortmannin (class I, class II, and class III PI3K inhibitor). Compared to dimethyl sulfoxide (DMSO) (D on the *x*-axis), none of these compounds caused major impacts on virus RNA counts at 6 h post-infection, since any fluctuations in RNA levels never reached a twofold-change (58) (Fig. S3B through G). Only 3-MA and VPS34IN reduced virus RNA synthesis at concentrations much higher than their 50% inhibitory concentration (IC_50_) dose for autophagy inhibition (Fig. S3F and G). Importantly, no cytotoxicity was caused by either of these drugs at these concentrations (Fig. S3H), suggesting that their impact on virus RNA may be due to pleiotropic effects. Overall, our findings support that autophagy is dispensable for SARS-CoV-2 RNA synthesis.

### The SARS-CoV-2 RTC co-localizes with RAB5^+^ membranes

**Fig 3:**
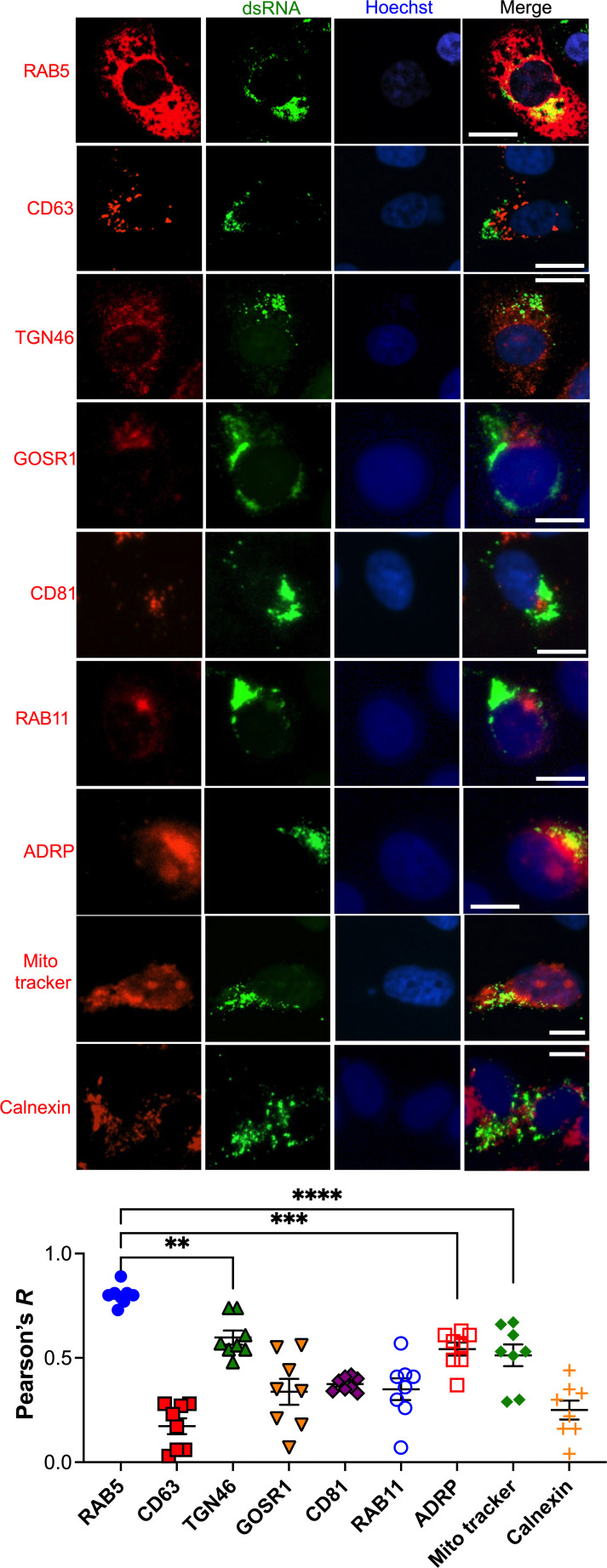
Early endosomal membranes highly co-localize with SARS-CoV-2 dsRNA. VeroE6 cells were infected with SARS-CoV-2 HK (MOI = 1) in a synchronized manner. Six hours later, cells were fixed, blocked, and stained for the virus dsRNA and the intracellular markers RAB5, CD63, TGN46, GOSR1, CD81, RAB11, ADRP, MitoTracker, and calnexin. Images: representative pictures of three independent experiments. White scale bar: 10 µm. Graph: the Pearson’s correlation coefficient (*R*) value for the co-localization of SARS-CoV-2 dsRNA and the intracellular markers listed above was calculated from eight randomly selected fields. Raw values with their mean and SEM are represented. **, *P* < 0.01; ***, *P* < 0.001; ****, *P* < 0.0001.

After ruling out a role for autophagy in RO formation, we examined the subcellular distribution of the virus RTC relative to several intracellular membrane markers. The reasoning is that the presence of cellular proteins on the RO surface could be indicative of their membrane origin. For this, we took an unbiased approach and used markers for several intracellular membranes, including early endosomes (RAB5), late endosomes (CD63), recycling endosomes (RAB11), *cis*-Golgi (GOSR1), *trans*-Golgi (TGN46), exosomes (CD81), ER (calnexin), lipid droplets (ADRP), and mitochondria (MitoTracker). As a control, the distribution of these cellular markers in the absence of infection was also examined (Fig. S5). VeroE6 cells were infected with SARS-CoV-2 HK (MOI = 1), and 6 h later, cells were stained for the virus dsRNA and the intracellular markers specified above. Little co-localization was observed between the virus dsRNA and recycling endosomes, exosomes, late endosomes, and the ER markers, with Pearson’s correlation coefficients between 0.1 and 0.3. Some degree of co-localization was detected with Golgi (TGN46), lipid droplets, and mitochondrial markers, with Pearson’s values between 0.5 and 0.6. However, the strongest co-localization was observed with the early endosomal marker (RAB5), with an average Pearson’s correlation coefficient of 0.8 (Fig. 3), which was significantly higher than the co-localization observed for TGN46, ADRP, and mitochondria. Similar results were obtained when infected cells were stained for the viral RdRp as a proxy for the RTC (Fig. S6). Remarkably, upon infection, we noticed that some of these intracellular markers, especially RAB5 and TGN46, appear more intense and dispersed throughout the cytosol, which likely reflects that the infection is triggering these changes (Fig. S7). In fact, it has been shown that SARS-CoV-2 promotes Golgi fragmentation, reshapes the cytoskeleton, and causes *RAB5* mRNA upregulation in COVID-19 patients (31, 59, 60), so our findings are in line with those reports. Overall, our assays reveal that the virus RTC primarily overlaps with RAB5, suggesting that the virus uses endosomal membranes to build ROs or that it recruits RAB5 to the RO membrane.

### RAB5^+^ early endosomes are required for the biogenesis of SARS-CoV-2 ROs

**Fig 4:**
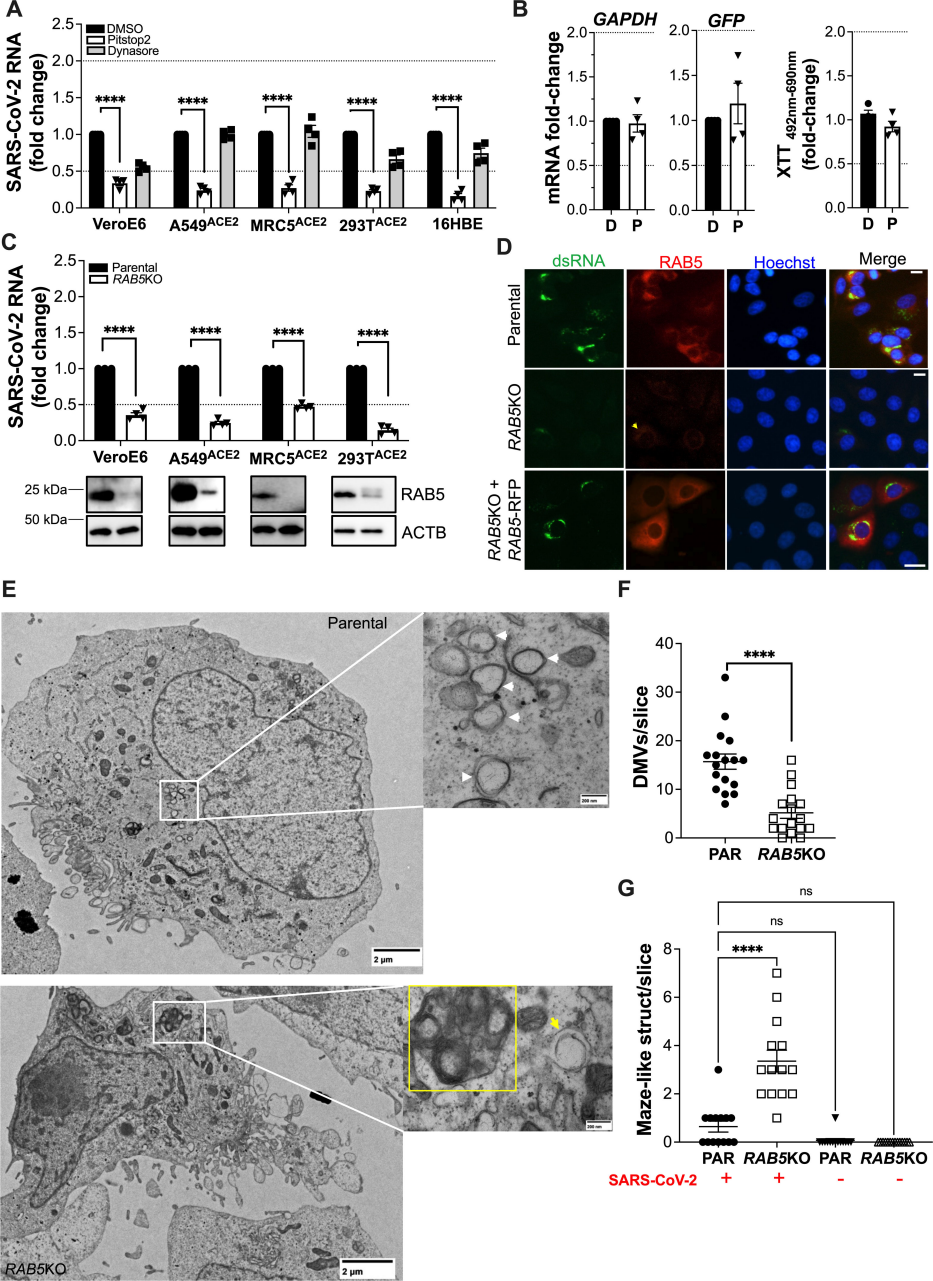
Clathrin and RAB5 are necessary for SARS-CoV-2 RNA synthesis and RO formation. (A) VeroE6, A549-ACE2, MRC5-ACE2, HEK293T-ACE2, and 16HBE cells were infected with SARS-CoV-2 HK (MOI = 1) in a synchronized manner. 4.5 h post-infection (8.5 h for 16HBE), cells were incubated with DMSO, pitstop2 (100 μM), or dynasore (80 μM) for 1.5 h. Their effect on virus RNA synthesis was examined by RT-qPCR, using primer pairs for *NSP12* and *NSP6*. SARS-CoV-2 RNA levels were expressed as fold-change after normalization with DMSO-treated cells. (B) The effect of DMSO (D) and pitstop2 (P) on mRNA levels of *GAPDH* or ectopic DNA (GFP provided by transfection) was investigated by adding 100 μM of the drug to VeroE6 cells for 1.5 h. Cells were harvested, RNA extracted, converted to cDNA, and analyzed by qPCR. Data are expressed as fold-change over DMSO. The impact of 6 h incubation of pitstop2 on cell viability was assessed by XTT assays. Data are expressed as fold-change over DMSO. Data correspond to the mean and SEM of four independent experiments. (C) Parental and *RAB5*KO VeroE6, A549-ACE2, MRC5-ACE2, and HEK293T-ACE2 cells were infected with SARS-CoV-2 HK (MOI = 1) in a synchronized manner. Six hours post-infection, total RNA was collected. SARS-CoV-2 genome copy numbers were determined by RT-qPCR using primer pairs for *NSP12* and *NSP6* and expressed as fold-change after normalization with the parental cells. Blots underneath confirm the depletion of RAB5. ACTB/β-actin is used as a housekeeping gene. Data correspond to the mean and SEM of four independent experiments. (D) Representative images of parental VeroE6, VeroE6 *RAB5*KO, and VeroE6 *RAB5*KO + RAB5 RFP cells infected with SARS-CoV-2 HK (MOI = 1) and stained 6 h post-infection for the virus dsRNA (green), RAB5 (red), and the nuclei (blue). White scale bar: 10 µm. (E) Parental VeroE6 (top) and *RAB5*KO VeroE6 cells (bottom) were infected with SARS-CoV-2 HK at MOI = 5 in a synchronized manner. Six hours later, cells were fixed for transmission electron microscopy processing. The presence of double membrane vesicles (DMVs) is depicted with white and yellow arrowheads. Areas delineated in white squares are shown at a higher magnification in the right panels. Incomplete DMVs are found within yellow squares. Scale bars: 2 µm and 200 nm. Images are representative of three independent experiments. (F, G) The quantification of DMVs per cell (F) as well as maize-like structures (G) for >14 cells is provided for each experimental condition (parental and *RAB5*KO cells). Raw values with their mean and SEM are represented. ****, *P* < 0.0001; ns, not significant.

Because RAB5 is the marker that most strongly co-localized with the virus RTC, and this strong co-localization was corroborated in the other cells used in this study (Fig. S8), we explored the role of early endosomes in SARS-CoV-2 genome replication. For this, we used the inhibitors pitstop2 and dynasore. Pitstop2 inhibits clathrin oligomerization with adaptor proteins, which impacts the maintenance of the early endosomal population by interfering with vesicular transport from the plasma membrane and Golgi (61–64). Dynasore inhibits dynamins, which are involved in fission events during receptor-mediated endocytosis. Hence, the use of pitstop2 and dynasore could help us understand what process of early endosome biogenesis is important for SARS-CoV-2 genome replication. Importantly, besides fusion at the plasma membrane, SARS-CoV-2 can enter target cells by receptor-mediated endocytosis, and the selection between these two modes of entry is dictated by the presence of transmembrane serine protease 2 (TMPRSS2) on the cell surface as well as mutations accumulated in the Spike of certain SARS-CoV-2 variants (65–68), which favor fusion at endosomes. Although the HK variant uses membrane fusion as the entry mechanism (65), we wanted to exclude any impact these inhibitors might have on entry, which would consequently affect virus RNA synthesis. Since our kinetics assay showed that new genome copies are detectable by 6 h post-infection (Fig. 1A), drugs were added 4.5 h after synchronized infection, so we could influence RO formation without impacting entry. Consistent with the distribution of the virus RTC at RAB5^+^ early endosomes, pitstop2 significantly reduced virus RNA synthesis. However, dynasore had only a minor impact (Fig. 4A), which varied across cell lines, suggesting that endosomes from endocytic vesicles play a secondary role in SARS-CoV-2 RNA synthesis. To rule out that pitstop2’s impact on virus RNA is not due to toxicity, the effects of this compound on host transcription (*GAPDH* mRNA), RNA synthesis from ectopic DNA [green fluorescent protein (GFP)-coding plasmid], and cell viability were assessed by RT-qPCR and XTT assays. These assays showed no defects in host or ectopic mRNA synthesis nor evidence of cytotoxicity (Fig. 4B).

The role of RAB5 in SARS-CoV-2 RNA synthesis was confirmed by generating *RAB5* knockout cells. To assess for any effects that deleting *RAB5* might exert on virus entry, we examined for differences in virus RNA at 2 and 6 h post-synchronized infection between parental and *RAB*5KO cells. As for the *ATG5*KO assays, qPCR data are expressed as the inverse value of the raw Cq (1/Cq), but these data are also presented as fold-change over parental cells in Fig. S4B. Deletion of *RAB5* did not delay virus entry, since virus RNA counts in *RAB5*KO cells were similar or higher than parental cells at 2 h post-infection (Fig. S4B and S9A). However, deletion of *RAB5* significantly impaired SARS-CoV-2 RNA synthesis in all cells investigated (Fig. 4C; Fig. S4B and S9A, black circles). To further exclude a possible impact of deleting *RAB5* on virus replication due to a defect in virus entry, we used an entry reporter assay. For this, we infected parental and *RAB5*KO HEK293T-ACE2-30F-PLP2 cells with SARS-CoV-2 HK at MOIs = 2 and 5, and cells were analyzed 4 h post-infection. HEK293T-ACE2-30F-PLP2 cells are engineered to constitutively express renilla luciferase and a SARS-CoV-2 PLpro-activatable firefly luciferase, which allow to measure infectivity (69) (Fig. S9B, cartoon). Because SARS-CoV-2 PLpro is a non-structural protein encoded within *ORF1ab*, which is expressed without the need of generating ROs, this cell line allows us to measure virus entry and whether knocking out *RAB5* has an impact on it. No differences in firefly luciferase activation were observed between parental and *RAB5*KO cells, regardless of the MOI used (Fig. S9B), further supporting that RAB5 is dispensable for SARS-CoV-2 HK entry but is required for the replication of its genome. These quantitative measurements are empirically supported by fluorescence microscopy, where *RAB5* deletion caused a severe loss in products of virus genome replication like dsRNA. In fact, the only cells with dsRNA staining happened to have some residual RAB5 expression (Fig. 4D, yellow arrowhead). Accordingly, reconstitution of RAB5 into *RAB5*KO cells rescued the phenotype (Fig. 4D, bottom). To confirm a role for RAB5 in the formation of ROs, transmission electron microscopy (TEM) images were obtained from parental and *RAB5*KO VeroE6 cells. For this, cells were infected with SARS-CoV-2 HK at MOI = 5. Six hours later, cells were processed for TEM along with uninfected cells to confirm that juxtaposed DMVs are only observed under conditions of infection (Fig. S10). Unlike the infected parental cells, where several DMVs adjacent to each other were observed (Fig. 4E, top, white arrowheads), *RAB5*KO-infected cells displayed notably less DMVs (Fig. 4E, bottom, yellow arrow), and many maze-like membranous structures (Fig. 4E, bottom, yellow square), suggesting initiation of membrane remodeling, but failure to generate ROs. Quantification of DMVs and maze-like structures from 14 to 17 cells per experimental condition confirmed that deletion of *RAB5* causes a significant defect in RO formation and ruled out possible membrane artifacts due to deleting *RAB5* (Fig. 4F and G). Overall, our results indicate that clathrin-dependent RAB5^+^ early endosomes are required for SARS-CoV-2 genome replication and RO biogenesis.

### COPB1 is necessary for SARS-CoV-2 RNA synthesis

**Fig 5:**
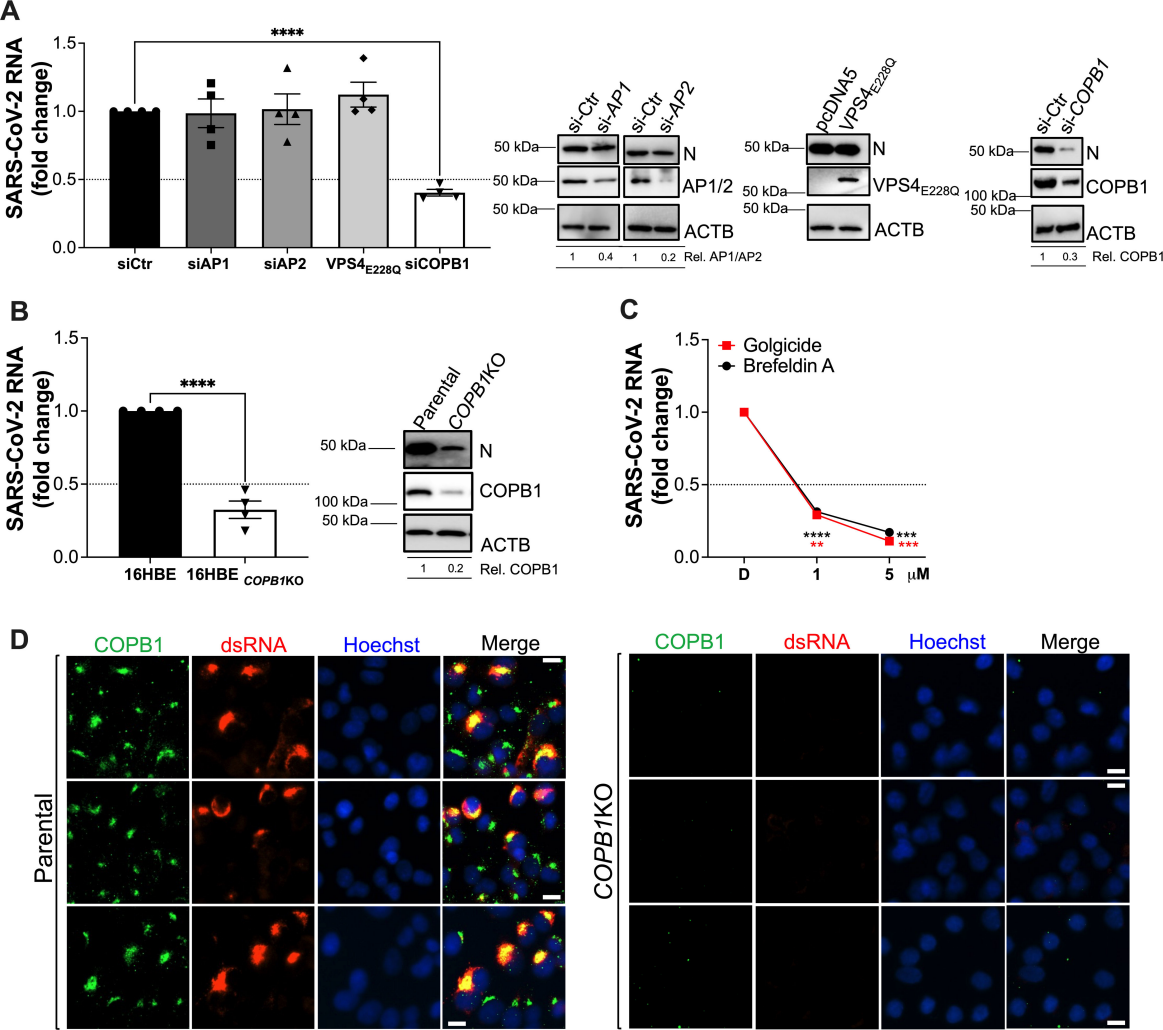
COPB1 is required for SARS-CoV-2 RNA synthesis. (A) HEK293T-ACE2 cells were transfected with siRNAs for AP1, AP2, and COPB1. To block the ESCRT machinery, cells were transfected with the VPS4 dominant-negative mutant VPS4_E228Q_. The effect of depleting or blocking these vesicular trafficking molecules on SARS-CoV-2 RNA synthesis was determined by RT-qPCR using primer pairs for *NSP12* and *NSP6* and expressed as fold-change after normalization with the siRNA control. For VPS4_E228Q_, data were normalized with an empty vector. Right panel shows representative blots to confirm the depletion or expression of the constructs indicated above. ACTB was used as a housekeeping gene. The impact of the depletion/expression of these constructs on the levels of SARS-CoV-2 late genes (i.e., nucleocapsid, N) was also examined. Relative expression levels are provided underneath the blots. (B) Parental 16HBE and *COPB1*KO cells were infected with SARS-CoV-2 HK (MOI = 1) in a synchronized manner. Ten hours post-infection, cells were harvested, and total RNA was collected. SARS-CoV-2 genome copy numbers were determined by RT-qPCR and expressed as fold-change after normalization with the parental cells. (C) The effect of COP-I inhibitors, golgicide and brefeldin A, on SARS-CoV-2 RNA synthesis was investigated in 16HBE cells after synchronized infection with SARS-CoV-2 HK (MOI = 1). Cells were treated 2 h post-infection at the indicated concentrations for 8 h prior to RNA extraction. SARS-CoV-2 genome copy numbers were determined by RT-qPCR and expressed as fold-change after normalization with DMSO (D on the *x*-axis). Data correspond to the mean and SEM of four independent experiments. **, *P* < 0.01; ***, *P* < 0.001; ****, *P* < 0.0001. (D) Representative fluorescence microscopy pictures of parental and *COPB1*KO A549-ACE2 cells infected with SARS-CoV-2 and stained for COPB1 (green) and the virus dsRNA (red). White scale bar: 10 μm.

To examine the role of factors involved in the trafficking of early endosomes in SARS-CoV-2 RNA replication, we depleted AP1, AP2, and COPB1, a component of the COP-I complex, using silencing RNA (siRNA). We also used a dominant-negative mutant of VPS4 (VPS4_E228Q_), an essential molecule for the function of the ESCRT (endosomal sorting complexes required for transport) machinery (70–72). Because of their higher transfection efficiency, here, we infected HEK293T-ACE2 cells that were previously transfected with these siRNAs and/or constructs. Depletion of these trafficking components as well as the expression of VPS4_E228Q_ were verified by western blot (Fig. 5A, blots). While depletion of AP1 and AP2 or blocking the ESCRT machinery had no major impact on SARS-CoV-2 RNA synthesis, the depletion of COPB1 caused a significant defect in virus genome replication (Fig. 5A). These findings were reproduced in 16HBE *COPB1*KO cells (Fig. 5B). To exclude any potential effects that knocking out *COPB1* might have on virus entry, virus RNA counts were compared between parental and *COPB1*KO 16HBE cells at 6 and 10 h post-infection, since RNA synthesis is not detected until 10 h post-infection in these cells. No differences in total RNA counts were observed at 6 h post-infection, reflecting no defects in entry (Fig. S4C and S9C). This was also corroborated in *COPB1*KO HEK293T-ACE2-30F-PLP2 cells (Fig. S9D). In contrast, a significant reduction in total RNA counts was detected at 10 h post-infection (Fig. S4C and S9C), further supporting that COPB1 is important for SARS-CoV-2 RNA synthesis. In line with this, 16HBE cells treated with brefeldin A or golgicide, inhibitors of the COP-I complex (73–77), showed a drastic reduction of SARS-CoV-2 RNA counts (Fig. 5C). These observations were empirically confirmed by fluorescence microscopy, where deletion of *COPB1* dramatically impacted the accumulation of virus RNA replication products (dsRNA, Fig. 5D). Therefore, these assays confirm a role for the COP-I machinery in the replication of the SARS-CoV-2 genome.

### SARS-CoV-2 NSP6 associates with RAB5 and COPB1

**Fig 6:**
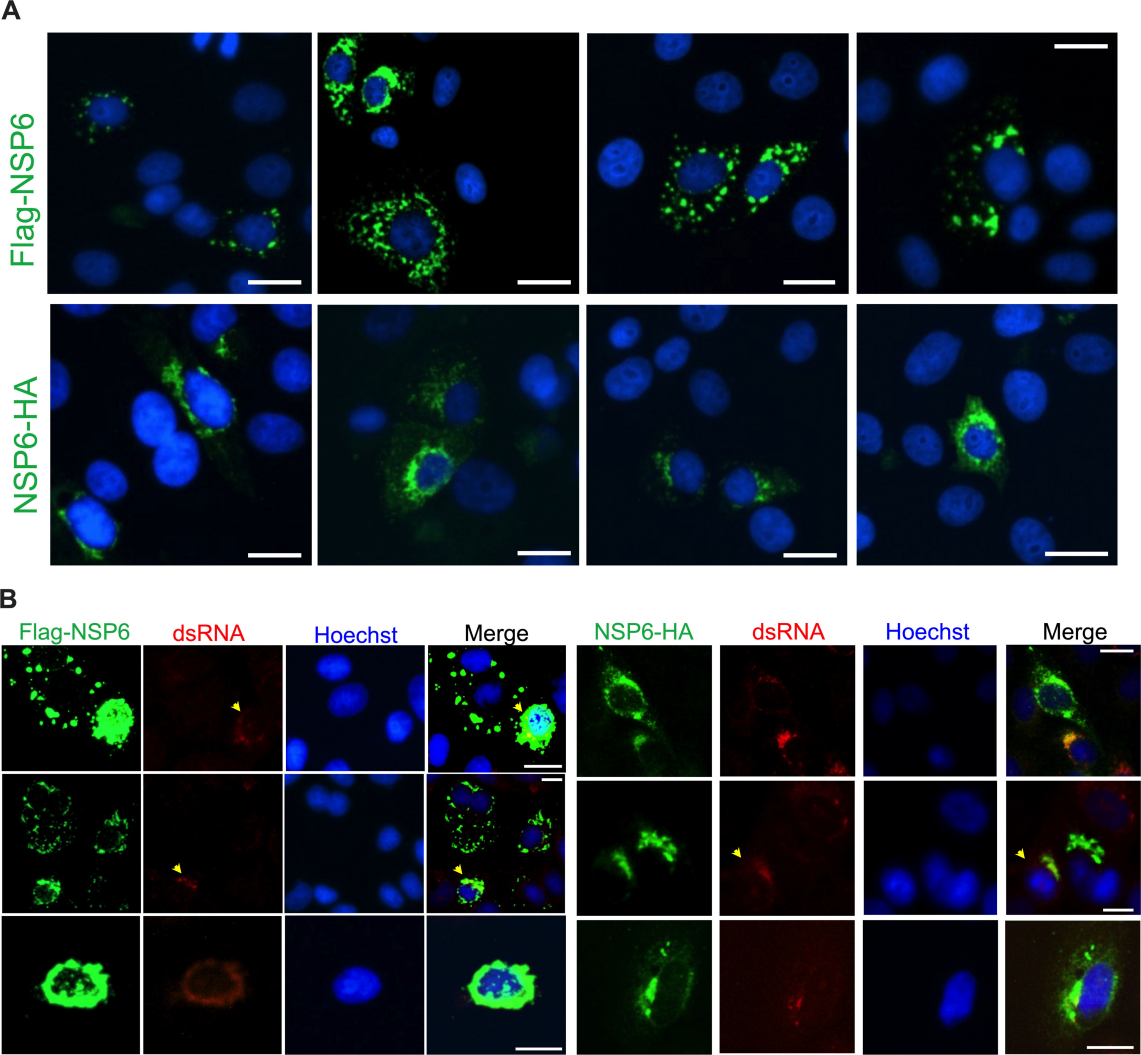
Tag location within NSP6 changes its distribution pattern, but its localization is restored in the context of infection. (A) Cellular distribution of Flag-NSP6 (top) and NSP6-HA (bottom) was investigated by fluorescence microscopy in VeroE6 cells. Cells are stained for Flag or HA (green) and the nucleus (blue). (B) VeroE6 cells were transfected with either Flag-NSP6 (left) or NSP6-HA (right) 1 day before infection with SARS-CoV-2 HK (MOI = 1). NSP6 distribution pattern was examined by fluorescence microscopy 6 h post-infection. Cells are stained for Flag or HA (green), the virus dsRNA (red), and the nucleus (blue). Yellow arrowheads indicate infected cells. White scale bar: 10 μm. Images are representative of three independent experiments.

Together with NSP3 and NSP4, NSP6 is an ER, multipass transmembrane coronavirus protein critical for the creation and maintenance of replication organelles (9–13). In fact, the sole co-expression of NSP3, NSP4, and NSP6 induces membrane remodeling and DMVs as the ones observed during coronavirus infection (9, 10, 78, 79). However, in the absence of NSP3 and NSP4, NSP6 only induces membrane zippering, and these rearrangements are not sufficient to trigger RO formation (10, 16). Importantly, a recent study found that when expressing tagged NSP6 in the absence of NSP3 and NSP4, the location of the tag (N-terminus vs C-terminus) can drastically affect its subcellular distribution (10). Consistent with that report, we found that N-terminally tagged NSP6 displays a puncta distribution (Fig. 6A, top), while C-terminally tagged NSP6 shows a reticular and perinuclear localization (Fig. 6A, bottom). This reticular distribution of NSP6 tagged at the C-terminus has already been observed for other coronaviruses, where it was concluded that NSP6 resides in the ER (27). In contrast, we found that NSP6 had a similar distribution phenotype regardless of where the tag was attached when expressed in infected cells (where NSP3 and NSP4 are present) (Fig. 6B), and this pattern is more similar to that of the C-terminally tagged NSP6 (Fig. 6A and B). Because NSP3 and NSP4 remodel cellular membranes that are later reorganized by NSP6 (10), one could envision a scenario where NSP6 is recruited to these sites to help in DMV organization, which likely contributes to the correct localization of the protein regardless of its tag location. Overall, these data further reinforce the need of NSP3/4/6 for successful RO formation.

**Fig 7:**
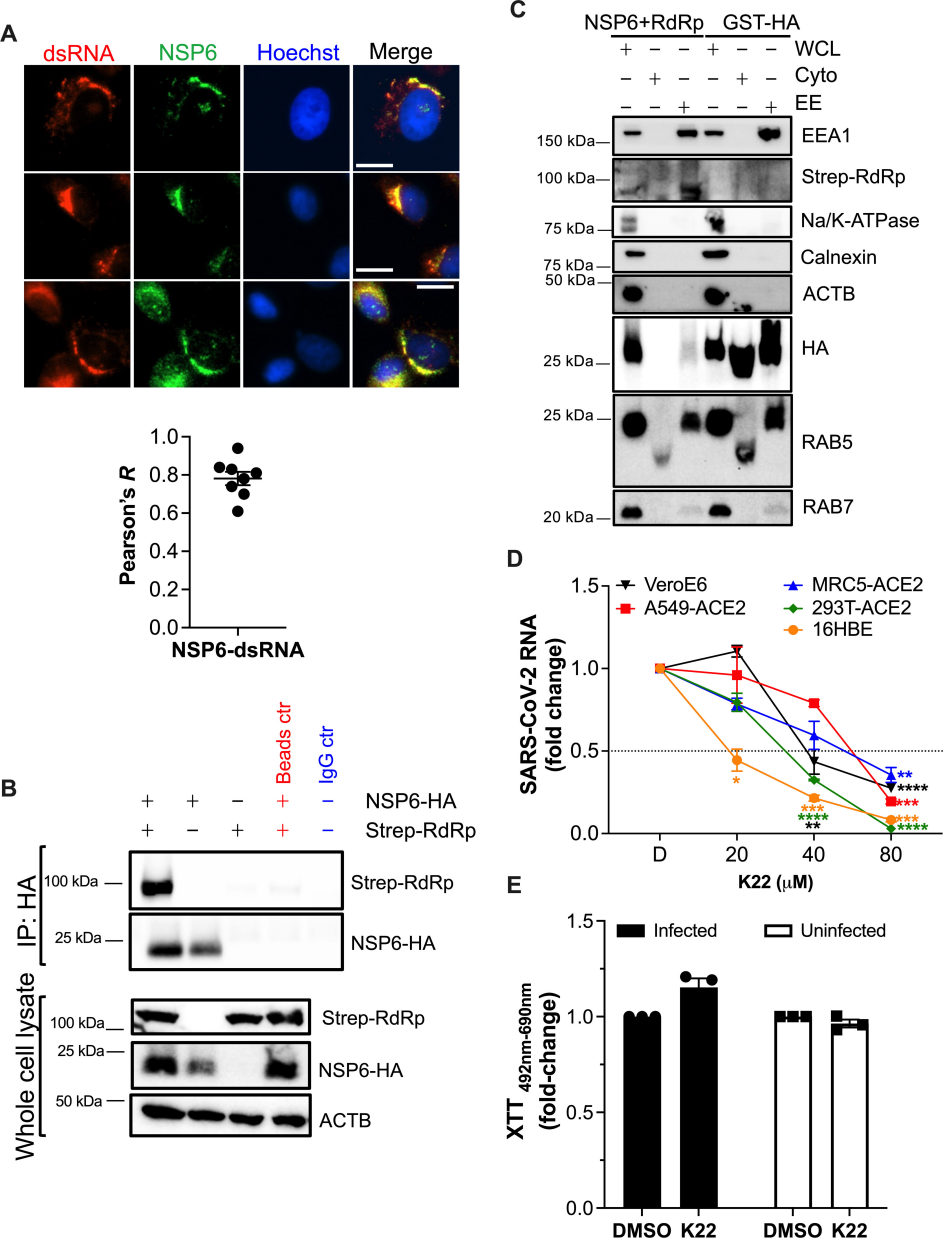
NSP6 co-localizes, interacts, and co-purifies with the virus RTC and is necessary for SARS-CoV-2 RNA synthesis. (A) VeroE6 cells were infected with SARS-CoV-2 HK (MOI = 1) in a synchronized manner. Six hours later, cells were fixed, blocked, and stained for the nuclei (blue), NSP6 (green), and dsRNA (red). Pictures are representative of three independent experiments. White scale bar: 10 µm. Graph: the Pearson’s correlation coefficient (*R*) value for the co-localization of dsRNA and NSP6 was calculated from eight randomly selected fields. Individual values, their mean, and SEM are shown. (B) HEK293T cells were co-transfected with constructs coding for NSP3, NSP4, NSP6-HA, and Strep-RdRp. Forty-eight hours later, lysates were immunoprecipitated against NSP6-HA, and membranes were probed for Strep-RdRp. Lysates were also analyzed for RdRp, NSP6, and ACTB. Data are representative of three independent experiments. (C) Similar transfections were performed to investigate the presence of the virus RTC and NSP6 in early endosomal membranes by subcellular fractionation. Glutathione S-transferase (GST) was used as a control present in the cytosol and endosomes. Purity of the fractions was confirmed using antibodies against specific early endosomal markers (RAB5, EEA1), late endosomal markers (RAB7), cytosolic markers (non-prenylated RAB5), plasma membrane markers (Na/K ATPase), ER markers (calnexin), and cytoskeleton markers (ACTB). Low presence of RAB7 in the early endosomal fraction is expected, as the transition from early to late endosomes requires a RAB cascade where RAB5 and RAB7 temporarily co-exist. WCL, whole cell lysate. EE, early endosomal fraction. Cyto, cytosolic fraction. Data are representative of three independent experiments. (D) VeroE6, A549-ACE2, MRC5-ACE2, HEK293T-ACE2, and 16HBE cells were infected with SARS-CoV-2 HK (MOI = 1) in a synchronized manner. Increasing concentrations of the NSP6 inhibitor K22 were added 2 h post-infection. Four hours later (8 h for 16HBE cells), cells were harvested, total RNA was extracted, and the SARS-CoV-2 genome copy numbers were determined by RT-qPCR using primer pairs for *NSP12* and *NSP6*. The effect of K22 on virus RNA synthesis was expressed as fold-change after normalizing to DMSO (D on the *x*-axis). (E) The impact of K22 at 80 μM on cellular viability and toxicity was examined by XTT assays in both infected and uninfected VeroE6 cells and is expressed as fold-change over DMSO. *, *P* < 0.05; **, *P* < 0.01; ***, *P* < 0.001; ****, *P* < 0.0001. Data correspond to the mean and SEM of three independent experiments.

Unlike NSP3 and NSP4, NSP6 is more amenable to visualization and detection (80), so we investigated its role in recruiting RAB5^+^ membranes. For this, our SARS-CoV-2 NSP6-HA construct is either co-transfected along with its NSP3 and NSP4 counterparts or transfected in infected cells to ensure proper subcellular distribution. Using this approach, we confirmed that both NSP6-HA and NSP6 produced during infection share a similar subcellular distribution, which co-localizes with the virus RTC (Fig. 6B and 7A, respectively). Accordingly, NSP6-HA co-immunoprecipitates with the virus RdRp (Fig. 7B), and in line with the extensive co-localization between the virus RTC and RAB5^+^ membranes, both NSP6-HA and the virus RdRp co-purify with the early endosomal fraction (Fig. 7C), further supporting that SARS-CoV-2 uses early endosomes to build ROs and that NSP6 distributes at these locations. The role of NSP6 in SARS-CoV-2 RNA synthesis has previously been confirmed using K22, a compound that specifically disables NSP6 (10, 15), and we further corroborated those findings showing that K22 causes a dose-dependent defect in virus RNA counts (Fig. 7D). Of note, XTT assays using 80 μM of K22 ruled out any cytotoxic effects (Fig. 7E).

**Fig 8:**
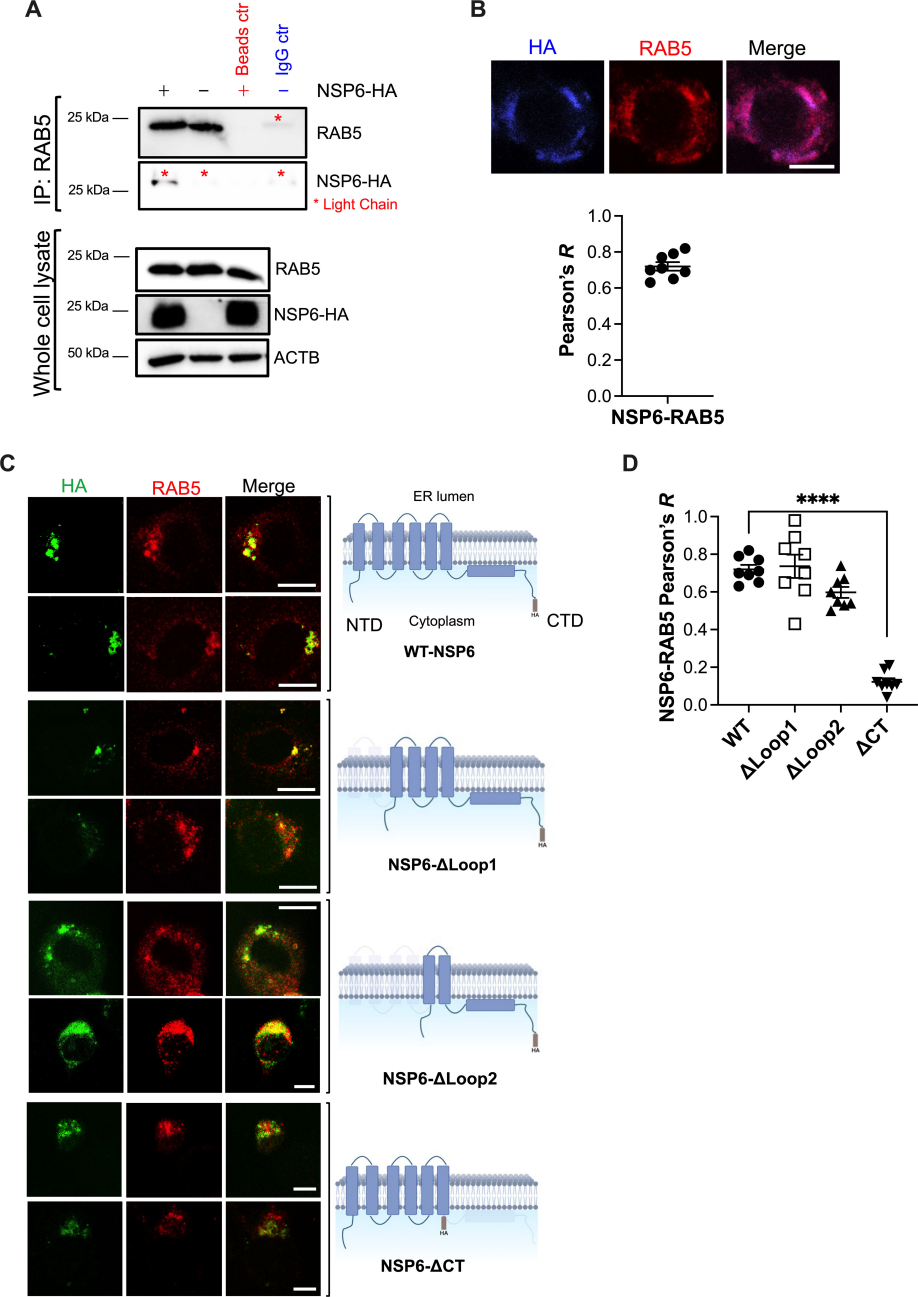
NSP6 interacts and co-localizes with RAB5. (A) HEK293T cells were co-transfected with NSP3, NSP4, and either NSP6-HA or an empty vector. Forty-eight hours later, cells were harvested, and RAB5 was immunoprecipitated. The pulldown fraction was examined for RAB5 and NSP6-HA. Lysates were also analyzed by western blot for RAB5, NSP6-HA, and ACTB. Blots are representative of three independent experiments. (B) VeroE6 cells were co-transfected with NSP6-HA, NSP3, and NSP4. Forty-eight hours later, cells were analyzed by fluorescence microscopy for the distribution of NSP6 relative to RAB5. Images are representative of three independent experiments. Graph: the Pearson’s correlation coefficient (*R*) value for the co-localization of RAB5 and NSP6 was calculated from eight randomly selected fields. Individual values, their mean, and SEM are shown. (C) VeroE6 cells were co-transfected with NSP3, NSP4, and either wild-type NSP6-HA or different truncation mutants of NSP6. Forty-eight hours post-transfection, cells were fixed, blocked, and stained for HA and RAB5 to assess their degree of co-localization. Images are representative of three independent experiments. White scale bar: 10 μm. Illustrations of NSP6 mutants were created with BioRender. (D) The Pearson’s correlation coefficient (*R*) value for the co-localization of RAB5 and the NSP6 constructs was calculated from eight randomly selected fields. Individual values, their mean, and SEM are represented. ****, *P* < 0.0001.

**Fig 9:**
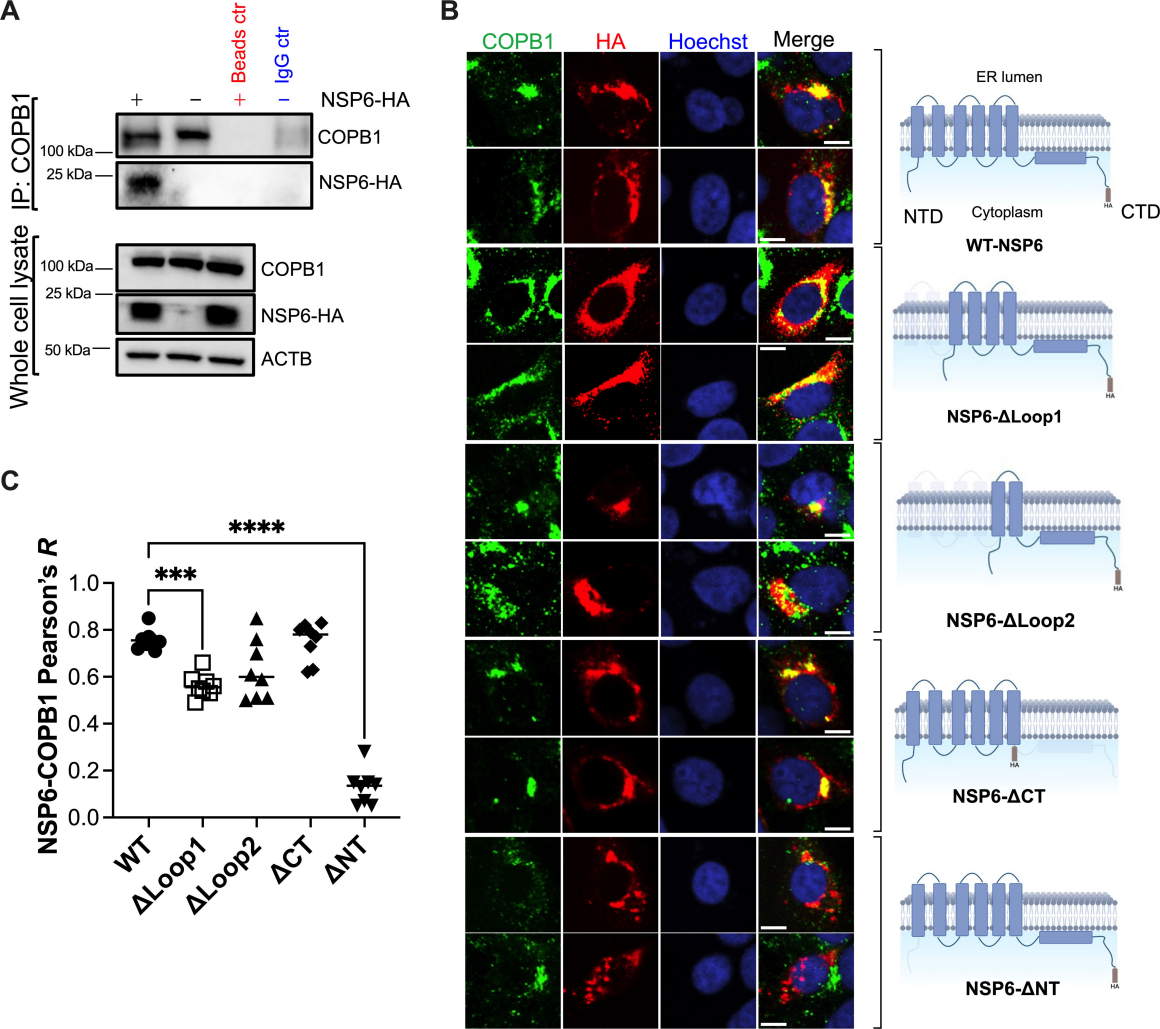
NSP6 interacts and co-localizes with COPB1. (A) HEK293T cells were co-transfected with NSP3, NSP4, and either NSP6-HA or an empty vector. Forty-eight hours later, cells were harvested, and COPB1 was immunoprecipitated. The pulldown fraction was examined for COPB1 and NSP6-HA. Lysates were also analyzed by western blot for COPB1, NSP6-HA, and ACTB. Blots are representative of three independent experiments. (B) VeroE6 cells were co-transfected with NSP3, NSP4, and either wild-type NSP6-HA or different truncation mutants of NSP6. Forty-eight hours post-transfection, cells were fixed, blocked, and stained for COPB1 and HA to assess their degree of overlap. Hoechst was included to label the nucleus. White scale bar: 10 µm. Images are representative of three independent experiments. (C) The Pearson’s correlation coefficient (*R*) value for the co-localization of COPB1 and the NSP6 constructs was calculated from eight randomly selected fields. Individual values, their mean, and SEM are represented. Illustrations of NSP6 mutants were created with BioRender. ***, *P* < 0.001; ****, *P* < 0.0001.

After confirming its relevance in SARS-CoV-2 RNA synthesis, we explored a potential role for NSP6 in recruiting RAB5 and COPB1 for the formation of ROs through immunoprecipitation (IP) and fluorescence microscopy of cells transfected with SARS-CoV-2 NSP6-HA or truncation mutants of this protein—along with NSP3 and NSP4. Here, NSP6 domains facing the cytoplasm (the regions more likely to interact with RAB5 and/or COPB1) were removed. We found that NSP6 co-immunoprecipitates with RAB5 (Fig. 8A) and distributes at RAB5^+^ locations (Fig. 8B), and that deletion of the C-terminal domain of NSP6 causes its mislocalization away from RAB5 (Fig. 8C and D), suggesting that the C-terminus of NSP6 is important for its association with RAB5. Similarly, NSP6 was found to co-immunoprecipitate with COPB1 (Fig. 9A). In this case, the NSP6 N-terminus seems to be involved in this interaction, since its truncation causes NSP6 to localize away from COPB1 (Fig. 9B and C). Importantly, our data do not exclude the participation of NSP3 and NSP4 in RAB5 and COPB1 recruitment, and given their concerted actions with NSP6, it is likely that they are also involved to some extent. Also, while our immunofluorescence images indicate that NSP6 uses its C-terminus to co-localize with RAB5 and that it uses its N-terminus to co-localize with COPB1, this technique does not have the resolution to assess protein-protein interactions. We tried to address this by co-immunoprecipitation. Unfortunately, some of the NSP6 truncation mutants were unspecifically being pulled down in their respective bead controls, so we could not reliably make conclusions about their binding with RAB5 or COPB1.

**Fig 10:**
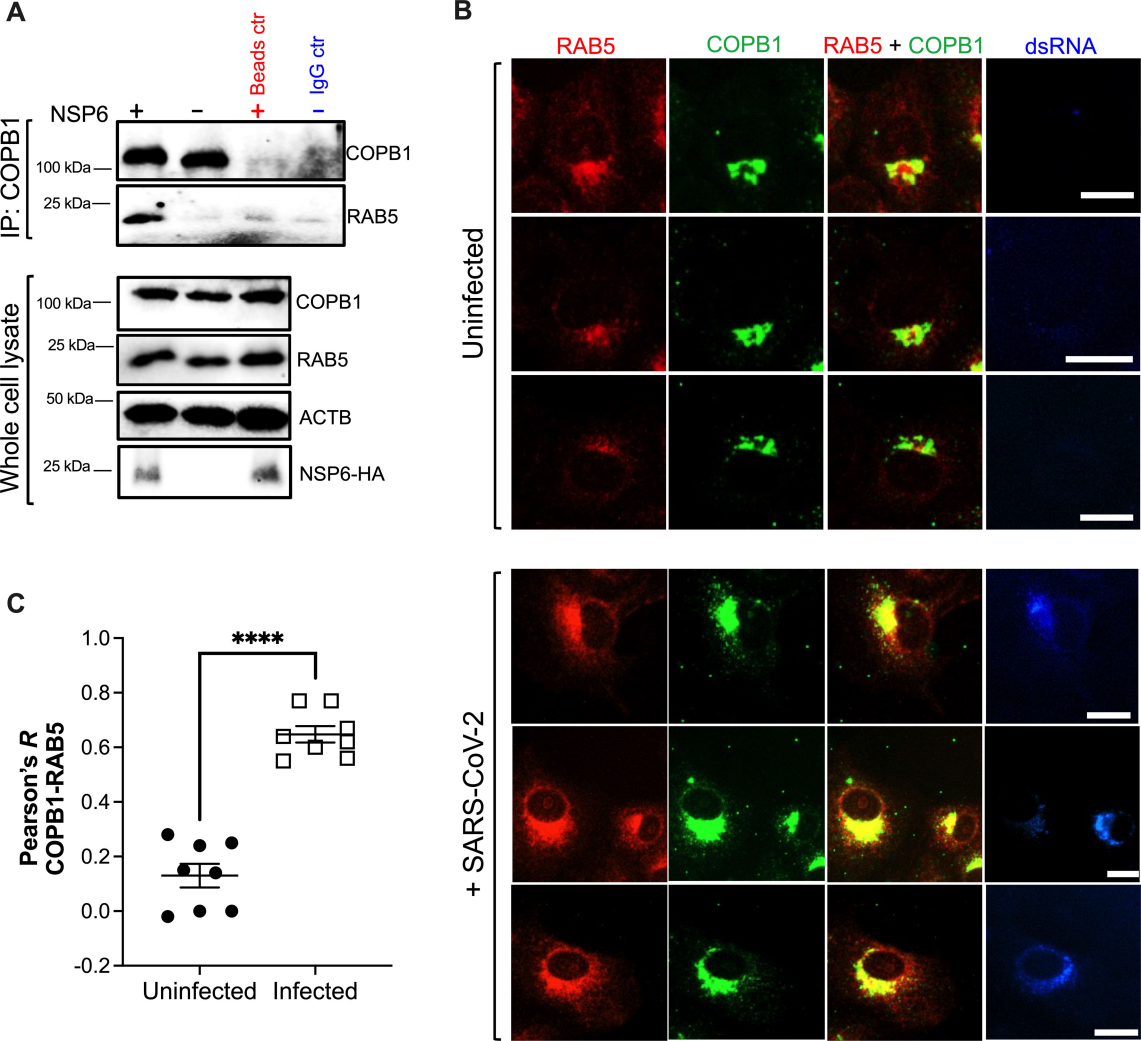
NSP6 enhances an association between RAB5 and COPB1. (A) HEK293T cells were co-transfected with NSP3, NSP4, and either NSP6-HA or an empty vector. Forty-eight hours later, cells were harvested, and COPB1 was immunoprecipitated. The pulldown fraction was examined for COPB1 and RAB5. Lysates were also analyzed by western blot for COPB1, NSP6-HA, RAB5, and ACTB. Blots are representative of three independent experiments. (B) VeroE6 cells were infected with SARS-CoV-2 HK (MOI = 1). Uninfected cells were included as control. Six hours later, cells were stained for RAB5 (red) and COPB1 (green). dsRNA (blue) was included to stain for infected cells. White scale bar: 10 µm. Images are representative of three independent experiments. (C) The Pearson’s correlation coefficient (*R*) value for the co-localization of COPB1 and RAB5 was calculated from eight randomly selected fields. Individual values, their mean, and SEM are represented. ****, *P* < 0.0001.

Finally, we used co-IP to explore whether NSP6 enhances an association between COPB1 and RAB5. While there seems to be minimal binding between these two proteins in the absence of NSP6, NSP6 enhanced their interaction (Fig. 10A). In line with this result, their degree of co-localization was significantly higher in infected cells (Fig. 10B and C). Overall, our findings indicate that RAB5^+^ membranes are a source for SARS-CoV-2 ROs and that NSP6 participates in their recruitment, likely by facilitating an association between RAB5 and COPB1.

## DISCUSSION

SARS-CoV-2 is the seventh coronavirus infecting humans. As for other human coronaviruses, SARS-CoV-2 likely emerged from a bat coronavirus (81–83) and quickly spread among the human population, suggesting that, besides finding an appropriate receptor for entry and being capable of evading/antagonizing the human antiviral defenses, the virus needed little adaptation after the spillover event. A possible explanation might be that the virus utilizes cellular factors that are widely distributed across mammals (84, 85), such as those hijacked for the biogenesis of ROs. ROs are common for all positive-sense RNA viruses. They are membranous structures that serve as platforms for the assembly of the virus RTC (43, 85). In the case of coronaviruses, ROs take the form of DMVs, which, besides aiding in genome replication and transcription, also conceal intermediate products of RNA synthesis (i.e., dsRNA) away from innate sensors (12, 14, 86). Therefore, ROs represent critical structures for coronavirus replication. Because the formation of ROs needs to occur before the replication of the virus genome (11, 31, 48), an emerging coronavirus that is adapting to a new host should be able to utilize the cellular machinery to create ROs. Otherwise, the virus polymerase cannot synthesize new genome copies and simultaneously introduce mutations that would further aid in the adaptation to the new species. Following this rationale, we hypothesized that SARS-CoV-2 uses a conserved cellular process to build ROs, and that the use of such common pathway enabled its transmission across hosts. Uncovering the underlying mechanism by which SARS-CoV-2 creates ROs not only will increase our understanding of the biology of this virus, but also can reveal new targets to treat COVID-19 as well as uncover dependency factors common to other coronaviruses.

Autophagy is an evolutionarily conserved pathway (87) that has been extensively studied for its potential role in RO biogenesis (27), since the DMVs generated during coronavirus infections structurally resemble autophagosomes. In fact, those studies revealed that some coronaviruses usurp autophagic membranes to build ROs (23–30, 88, 89). However, recent reports have shown that autophagy is unnecessary for the assembly of the SARS-CoV-2 RTC (10, 30). Consistent with these findings, no overlap between autophagosome, phagophore, omegasome, or EDEMosome markers and the virus RTC was observed during SARS-CoV-2 RNA replication. Furthermore, deletion of *ATG5*, a gene essential for the formation of autophagosomes (54–57), had no effect on SARS-CoV-2 RNA synthesis. A role for autophagic elements was further excluded by using drugs that modulate this pathway. None of the compounds tested impacted virus genome replication. Only at very high concentrations, the class III PI3K inhibitors 3-MA and VPS34IN downregulated virus RNA. However, we cannot conclude that inhibition of class III PI3K through its role in autophagy accounts for the reduction of SARS-CoV-2 RNA synthesis, since this kinase has pleiotropic effects on other pathways, such as early endosome to late endosome transport, endocytosis, and phosphatidylinositol biosynthetic processes (90–92). Thus, our results are in line with a growing body of evidence that indicates that autophagy is dispensable for the assembly of the SARS-CoV-2 RTC (10, 30).

With the goal of identifying the membranes where SARS-CoV-2 assembles ROs, we examined the subcellular distribution of the virus RTC relative to intracellular membrane markers. Our assays revealed that the virus replication machinery highly overlaps with early endosomes, although some degree of co-localization was also found with Golgi, lipid droplets, and mitochondria. SARS-CoV-2 has been reported to cause extensive disorganization of the Golgi apparatus. In fact, we observed changes in the Golgi in infected cells. However, rather than contributing to the formation of ROs, Golgi fragmentation has been found to provide lipids for RO elongation and facilitate virion trafficking (59, 93). The role of lipid droplets in SARS-CoV-2 infection has recently been explained as a result of the vast membrane rewiring and metabolic reprogramming observed in infected cells (10, 94–96). Similarly, the observation that the SARS-CoV-2 RTC is in close proximity to mitochondria is not novel (97–99), and suggests that the virus positions ROs close to organelles that supply energy and metabolites for the formation and enlargement of its replication factories (31, 100). Despite the partial overlap with Golgi, lipid droplets, and mitochondria, we focused on investigating the role of RAB5 and early endosomes in the replication of the SARS-CoV-2 genome because RAB5 is the marker that most strongly and consistently co-localized with the virus replication machinery. Importantly, RAB5 has already been recognized as an important host dependency factor for SARS-CoV-2 entry (101, 102). SARS-CoV-2 can enter target cells by fusion at the plasma membrane or at endosomal membranes, and this is dependent upon the presence of TMPRSS2 on the cell surface. However, mutations accumulated in the Spike of SARS-CoV-2 variants have resulted in strains that favor fusion at endosomal membranes (65–68). For these strains, RAB5 is an essential factor (102). Because here we used a strain that relies on TMPRSS2 for entry, we were able to identify another critical role of RAB5 in SARS-CoV-2 replication.

Our studies demonstrate that RAB5 is crucial to build ROs. This may be explained by two scenarios: either the virus repositions RAB5 to RO membranes or the virus uses early endosomes (RAB5^+^ membranes) to generate ROs. To discriminate between these two possibilities, we used the endosomal inhibitors pitstop2, a clathrin inhibitor, and dynasore, a dynamin inhibitor, and examined their impact on SARS-CoV-2 genome replication. Clathrin is fundamental for the maintenance of the cellular early endosomal population by constant vesicular transport from the plasma membrane and other organelles, where Golgi is critical in this process (63, 64). However, dynamins only participate in endosome formation from the plasma membrane (103). Our assays revealed that SARS-CoV-2 needs clathrin to facilitate virus genome replication, while dynamins play a secondary role. Hence, these findings suggest that SARS-CoV-2 needs early endosomes (not just RAB5) for RNA synthesis, and that endocytic vesicles are not the primary source.

The requirement of RAB5^+^ endosomes for SARS-CoV-2 genome replication was confirmed in *RAB5* knockout cells, where (i) virus RNA synthesis was severely inhibited, (ii) the virus RTC was almost undetectable by fluorescence microscopy, and (iii) only few ROs were visible by electron microscopy. To understand how SARS-CoV-2 repurposes RAB5^+^ membranes for RO biogenesis, we studied the role of NSP6, since this protein is essential for the reorganization of DMVs to create functional ROs (9, 15, 104–107). In fact, a role for NSP6 in SARS-CoV-2 genome replication was confirmed using K22 (10, 15), an NSP6-specific inhibitor, which significantly reduced virus RNA synthesis. Our data confirmed that NSP6 co-localizes with the virus RTC. Furthermore, NSP6 and the RTC co-purify with the early endosomal fraction. This subcellular distribution likely explains NSP6 co-immunoprecipitation with the RdRp. However, whether this is a stable or a transient interaction is currently unknown. Our findings also suggest that NSP6 uses its C-terminal domain to interact with RAB5.

NSP6 is a multipass transmembrane protein found in the ER (104, 108, 109), away from endosomal membranes. So, how can this protein recruit early endosomes from the ER to generate ROs? A possible answer may be found in the fact that the endocytic pathway plays an important role in ER architecture and structure (110). Contact sites between the ER and endosomes have been reported to enable inter-organelle communication in a vesicular-independent manner and facilitate the exchange of lipids and small molecules (111–114). In addition to this molecular trade, membrane contacts between ER and endosomes are important for endosome fission, positioning, calcium exchange, and dephosphorylation of endosomal receptors (111, 115–122). In fact, it is estimated that 50% of early endosomes are in contact with the ER, and COP-I, a component of the retrograde transport from the Golgi apparatus to the ER, plays an important role in connecting endosomes to the ER (38, 39). Remarkably, we found that COPB1, which is part of the COP-I complex, is required to support SARS-CoV-2 genome replication, and previous studies with SARS-CoV-1 already identified COPB1 as a cellular factor that contributes to RO biogenesis (123–125). Therefore, it is likely that ER-bound NSP6 recruits endosomal membranes at these contact sites in a COP-I-dependent manner, and this is consistent with our *COPB1*KO assays, in which virus RNA synthesis is severely inhibited, and our immunoprecipitation and imaging studies showing not only NSP6-COPB1 and NSP6-RAB5 interactions, but also that NSP6 enhances an association between RAB5 and COPB1. Unlike its co-localization with RAB5, NSP6 co-localization with COPB1 requires the NSP6 N-terminus, which would theoretically allow NSP6 to interact with both molecules simultaneously. However, whether this interaction is direct and if the same NSP6 molecule is interacting with both COPB1 and RAB5 remains unclear.

Whereas the connection between the ER and early endosomes, and the implication of COPB1 in this process, helps understand how SARS-CoV-2 repurposes these membranes to create ROs, two questions remain unanswered: first, why does the virus use membranes from early endosomes instead of other endosomal structures? Second, does SARS-CoV-2 co-opt highly conserved cellular factors for RO biogenesis? For the first question, we could speculate that exploitation of early endosomes to create ROs allows for evasion of innate sensors. While endosomal Toll-like receptors (TLRs) are present in all endosomal membranes, for the most part, these pattern recognition receptors require pH-dependent proteolytic activation. Hence, by using membranes where TLR3 and TLR7/8 are less active, the virus genome, subgenomic RNAs, and dsRNA intermediates are less susceptible to being recognized by the innate sensing system (126–128)—offering the virus an advantage when parasitizing the cell. As for the second question, autophagy seemed a perfect candidate due to its evolutionary conservation and the creation of double membrane vesicles (autophagosomes). However, our findings indicate the endosomal pathway is critical for virus genome replication. Still, like autophagy, endocytosis and vesicular trafficking are widely distributed in metazoans with many components sharing a high degree of conservation within mammals (40). For instance, the clathrin heavy chain shares 100% identity within primates and 90% identity with rodents. Similarly, RAB5A shares 90% identity with that of canids, cattle, and rodents. COPB1 has 94% similarity with canids and bovine, and over 98% similarity with other primates. Hence, this high conservation, along with a potential selective advantage of using membranes with less active innate sensors, may have allowed SARS-CoV-2 to find an appropriate environment to support its replication in different mammalian species, which ultimately might have facilitated its propagation and adaptation to new hosts.

## MATERIALS AND METHODS

For detailed methods, refer to the supplemental material.

### Plasmids

Plasmids coding SARS-CoV-2 open reading frames (ORFs) were gifts from Dr. Konstantin Sparrer. HA-tagged full-length and truncated *NSP6* constructs were cloned into pcDNA5 (ThermoFisher Scientific, V601020). *ATG5*^KO^ cells were engineered using LentiCRISPRv2-*ATG5* (Addgene plasmid # 99573) (129). *COPB1*^KO^ and *RAB5*^KO^ cells were generated by transduction of LentiCRISPRv2 (Addgene plasmid # 52961) (130) harboring the following sgRNAs: *COPB1*: 5′-CAGGTTATCAAAGCGCTGAA-3′ and 5′-AGGTAGCACAAAACGAATGA-3′; *RAB5*: 5′-CGAGGCGCAACAAGACCCAA-3′ and 5′-GAGGCGCAACAAGACCCAAC-3′. psPAX2 (Addgene plasmid # 12260) and p-MD2-G (Addgene plasmid # 12259) were used to generate lentiviral particles to deliver Cas9 and sgRNAs, as well as to generate stable cell lines, following our previously reported protocols (131, 132). EGFP-LC3B was cloned into pQCXIP. pMX-EGFP-FYVE1 (Addgene plasmid # 38269) (133) was used to express DFCP1/FYVE1. Addgene plasmid # 80351 was used to express the dominant-negative mutant of VPS4, VPS4_E228Q_-EGFP (134). The Addgene plasmid # 14437 was used to express RAB5A fused with RFP (135).

### Cell culture and transfections

All cells were cultured in complete medium and supplemented with appropriate antibiotics and growth factors, following the manufacturer’s instructions as well as our previously established protocols (131, 132, 136). Cells were transfected using GenJet *in vitro* DNA transfection reagent (SignaGen Laboratories, SL100488) or Lipofectamine 3000 Transfection Reagent (ThermoFisher Scientific, L3000001), following the manufacturers’ instructions.

### Knockdown assays

esiRNA oligos were obtained to deplete *AP1*, *AP2*, and *COPB1* (ThermoFisher Scientific #138604, #14075, #s3373, respectively). A control siRNA SignalSilence was used (ThermoFisher Scientific, #6568). Knockdown of target genes was achieved by transient transfection of the esiRNAs in 10^6^ HEK293T-ACE2 cells using Lipofectamine 3000, following the manufacturer’s instructions. Knockdown was verified by western blot.

### XTT assay

XTT assays were performed in 2 × 10^4^-infected and uninfected VeroE6 cells in the presence of DMSO, 3-MA, VPS34IN, K22, or pitstop2, following our previously established protocol (137).

### SARS-CoV-2 infections

All infections were performed at the URMC Biosafety Level 3 (BSL3) laboratory, following the approved standard operating procedures.

Cells were infected with SARS-CoV-2 isolate Hong Kong (BEI Resources, NR52282; GenBank: MT547814) at MOI = 1, unless noted differently. Infections were kept at 4°C for 1 h for synchronization. Infected cells were maintained and processed as previously described (132). For RNA kinetics assays, cells were harvested at 2, 4, 6, 8, 10, 16, 24, and 48 h post-infection. In parallel, supernatants were collected for TCID_50_ assays. Similar infections were performed in the presence of siRNAs, cells depleted from ATG5, RAB5, or COPB1, or in the presence of drugs targeting autophagy, endocytosis, Golgi transport, or NSP6.

### Luciferase reporter assay

One million parental, *RAB5*KO, and/or *COPB1*KO HEK293T-ACE2-30F-PLP2 cells were infected with SARS-CoV-2 HK at MOIs 2 and 5. Four hours later, cells were harvested, and firefly and renilla luciferase activities were measured by luminescence using a microplate reader (BMG LABTECH, LUMIstar Omega). Uninfected cells were included for both parental and KO cells to subtract any background firefly luminescence. Data are presented as firefly luciferase over renilla luciferase.

### RT-qPCR

RNA extraction and cDNA synthesis were performed following our previously established protocol (131, 132, 137, 138). cDNA was used to measure the levels of SARS-CoV-2 RNA by qPCR using primers for NSP12 and NSP6, following our established qPCR method, including primers for quality control (131, 132, 137, 138). Changes in *NSP12* and *NSP6* RNA levels were determined by the 2^ΔΔCq^ method using *GAPDH* as the housekeeping gene. SARS-CoV-2 genome replication was calculated as fold-change over basal viral RNA. A fold-change of >2.0 or <0.5 relative to the control samples was considered biologically relevant (58).

### Median TCID_50_ assay

SARS-CoV-2 virus stocks and culture supernatants from infections were titrated by measuring their cytopathic effect by optical microscopy 3 days post-infection using the Spearman-Kärber method (139).

### Immunoprecipitation and western blotting

Infected, uninfected, and transfected cells were processed for immunoprecipitation and/or western blot following our previously established protocols, including handling of BSL3 samples (131, 132). Target proteins were pulled down using the primary antibodies listed in Table 1. Similarly, western blot membranes were probed with the primary and secondary antibodies listed in Table 1.

**TABLE 1:** Antibody sources and conditions

Protein	Primary antibody	Dilution	Source
ACTB/β-actin	Mouse monoclonal (8H10D10C4) to ACTB/β-actin	1:1,000	Cell Signaling Technology, 3700S
ADRP	Mouse monoclonal (2 C5A3) to ADRP	1:100	ThermoFisher Scientific, MA5-24797
ATG5	Rabbit polyclonal to ATG5	1:1,000	Cell Signaling Technology, 2630S
Calnexin	Mouse monoclonal (AF18) to CALNEXIN	1:100	ThermoFisher Scientific, MA3-027
CD63	Mouse monoclonal (MEM-259) to CD63 (LAMP-3)	1:100	ThermoFisher Scientific, MA1-19281
CD81	Mouse monoclonal (1.3.3.22) to CD81	1:100	ThermoFisher Scientific, MA5-13548
COPB1	Rabbit polyclonal to beta COP	1:1,000 1:100 for IP	ThermoFisher Scientific, PA1-061
dsRNA	Mouse monoclonal (rJ2) to dsRNA	1:200	Sigma-Aldrich, MABE1134
EDEM1	Rabbit polyclonal to EDEM1	1:100	Sigma-Aldrich, HPA029565
EEA1	Rabbit polyclonal to EEA1	1:1,000	Cell Signaling Technology, 2411
Flag	Rabbit monoclonal to DYKDDDDK	1:100	Cell Signaling Technology, 14793
GFP	Mouse monoclonal (4B10B2) to GFP	1:1,000	Sigma-Aldrich, SAB5300167
GOSR1	Mouse monoclonal (HFD9-5) to GOSR1	1:100	ThermoFisher Scientific, MA1-91008
HA	Mouse monoclonal to HA	1:1,000 for WB, 1:100 for flow cytometry, IP and microscopy	Biolegend, 901502
LC3B	Rabbit polyclonal to LC3B Rabbit monoclonal (D11) to LC3B	1:1,000 1:100	Cell Signaling Technology, 2775S Cell Signaling Technology, 3868
Mouse IgG	Goat anti-mouse (H + L) secondary HRP	1:2,000	ThermoFisher Scientific, 31430
Mouse IgG_1_	Rat anti-mouse IgG1 APC conjugated	1:100	ThermoFisher Scientific, 17–4015-82
Mouse IgG_1_	Goat polyclonal (Alexa-488 conjugated)	1:500	ThermoFisher Scientific, A-21121
Mouse IgG_1_	Goat polyclonal (Alexa-568 conjugated)	1:500	ThermoFisher Scientific, A-21124
Mouse IgG_2a_	Goat anti-mouse IgG_2a_ (Alexa-350 conjugated)	1:500	ThermoFisher Scientific, A-21130
Mouse IgG_2a_	Goat polyclonal (Alexa-488 conjugated)	1:500	ThermoFisher Scientific, A-21131
Mouse IgG_2a_	Goat polyclonal (Alexa-568 conjugated)	1:500	ThermoFisher Scientific, A-21134
Na/K ATPase	Rabbit polyclonal	1:1,000	Cell Signaling Technology, 3010
RAB11	Mouse monoclonal (1A12B9D8) to RAB11	1:100	ThermoFisher Scientific, MA5-31876
RAB5A	Mouse monoclonal (E6N8S) to RAB5A	1:1,000 for WB, 1:100 for microscopy and IP	Cell Signaling Technology, 46449
RAB7A	Rabbit polyclonal to RAB7A	1:1,000	Abcam, ab77993
Rabbit IgG	Goat anti-rabbit IgG (H + L), HRP conjugated	1:4,000	Promega, W4011
Rabbit IgG	Goat anti-Rabbit IgG (H + L) PE-Cy5.5	1:500	ThermoFisher Scientific, L42018
Rabbit IgG	Goat polyclonal (Alexa-488 conjugated)	1:500	ThermoFisher Scientific, A-11008
Rabbit IgG	Goat polyclonal (Alexa-568 conjugated)	1:500	ThermoFisher Scientific, A-11011
SARS-CoV-2 N	Rabbit monoclonal (ARC2372) to SARS-CoV-2 nucleoprotein	1:1,000	ThermoFisher Scientific, MA5-36086
SARS-CoV-2 NSP6	Rabbit polyclonal to SARS-CoV2-NSP6	1:100	ProSci, 9177
SARS-CoV-2 NSP12	Rabbit polyclonal to SARS-CoV-2 NSP12	1:100	GeneTex, GTX135467
SQSTM1/p62	Rabbit monoclonal (D1Q5S) to SQSTM1/p62	1:100	Cell Signaling Technology, 39749
Strep	Rabbit polyclonal to Strep-tag-II	1:1,000	Abcam, ab76949
TGN46	Mouse monoclonal (2 F7.1) to TGN46	1:100	ThermoFisher Scientific, MA3-063
WIPI2	Rabbit polyclonal to WIPI2	1:100	Abcam, ab229225

### Endosomal fractionation assay

HEK293T cells expressing SARS-CoV-2 NSP3, NSP4, NSP6-HA, and streptavidin-tagged RdRp (NSP12) were harvested, washed on ice-cold Dulbecco's phosphate buffered saline (DPBS), and lysed to obtain early endosomal and cytosolic fractions, following the Trident Endosome Isolation kit and instructions (GenTex, GTX35192). Whole cell lysate, cytosolic, and early endosomal fractions were analyzed by western blot for the presence of NSP6 (HA), RdRp (Strep), and the GST control (HA). Purity of the fractions was confirmed using antibodies against early endosomal markers (RAB5, EEA1), late endosomal markers (RAB7), cytosolic markers (non-prenylated RAB5), plasma membrane markers (Na/K ATPase), ER markers (calnexin), and cytoskeleton markers (ACTB) (see Table 1 for antibody specifications).

### Fluorescence microscopy

Infected, uninfected, and transfected cells were washed with DPBS, permeabilized, and fixed following our previously established protocol, including handling of BSL3 samples (132). Target proteins were visualized using antibodies listed in Table 1. The nuclei were stained with Hoechst (ThermoFisher Scientific, H3570; 1:5,000 dilution). Slides were imaged in a BioTek Lionheart FX automated microscope and a Nikon A1R HD with TIRF confocal microscope using 40× and 60×/1.49 oil objectives, respectively, and filter cubes/excitation diodes 350 nm, 488 nm, and 586 nm in order to excite 4',6-diamidino-2-phenylindole (DAPI), EGFP, and TexasRed, respectively. Images were processed and analyzed using the Gen5 software (BioTek Instruments, Winooski, VT). Proportional adjustments of brightness/contrast were applied.

### Electron microscopy

Parental and *RAB5*KO VeroE6 cells were infected with SARS-CoV-2 HK at an MOI = 5. As controls, uninfected parental and *RAB5*KO cells were included. Six hours later, cells were fixed in 2.5% glutaraldehyde and 4% paraformaldehyde in 0.1 M sodium cacodylate buffer and incubated for 24 h at 4°C. Next, cells were processed in the electron microscopy facility for thin sectioning at 70 nm. The sections were placed onto formvar/carbon copper slot grids and stained with aqueous uranyl acetate and 0.3% lead citrate. The grids were imaged using a Hitachi 7650 transmission electron microscope and an AMT 12 MP NanoSprint12 digital camera.

### Statistical analysis

Statistical calculations for two-group comparisons were performed with a two-tailed unpaired Student’s *t*-test. All other statistical comparisons were performed with one-way analysis of variance with Dunnett’s *post hoc* testing. Analyses were performed using Graph Pad Prism version 10.3.0. *P*-values ≤0.05 were considered statistically significant.

## Data Availability

All relevant data are in the article and its supplemental material. All reagents generated in this study are available from the lead contact upon request.

## SUPPLEMENTAL MATERIAL

Supplemental material mbio.03314-24-s0001.pdf Extended methods and supplemental figures.

ASM does not own the copyrights to Supplemental Material that may be linked to, or accessed through, an article. The authors have granted ASM a non-exclusive, world-wide license to publish the Supplemental Material files. Please contact the corresponding author directly for reuse.
