# Supplementary material for: RAB5 is a host dependency factor for the generation of SARS-CoV-2 replication organelles
Source: mBio. 2025 Apr 1;16(5):e03314-24. doi: 10.1128/mbio.03314-24 (PMC12077180; doi:10.1128/mbio.03314-24)
Supplement: Supplemental material — Extended methods and supplemental figures. [file mbio.03314-24-s0001.pdf]

EXTENDED MATERIALS AND METHODS

Plasmid DNA constructs.

(i) SARS-CoV-2 ORF constructs. Constructs coding for each SARS-CoV-2 ORF, including NSP3 and NSP4 were gifts from Dr. Konstantin Sparrer (Ulm University, Germany). SARS-CoV-2 *NSP6* as well as *NSP6* truncation mutants were cloned into the expression vector pcDNA5 (ThermoFisher Scientific, V601020) using the KpnI and BamHI unique restriction sites. Wild type and truncation NSP6 mutants harbored an HA tag in the C-terminus.

(ii) Lentivirus-based CRISPR-Cas9 genome editing constructs. ATG5 knockout cell lines were generated using LentiCRISPRv2-*ATG5*, a gift from Dr. Edward Campbell (Addgene plasmid # 99573)(129). *COPB1* and *RAB5* knockout cell lines were generated by CRISPR-Cas9. For this, the guide RNAs (gRNAs) that appear below were cloned into LentiCRISPRv2, a gift from Dr. Feng Zhang (Addgene plasmid# 52961)(130), using the BsmBI unique restriction site. Guide RNA sequences were generated using the CRISPR design tool (http://crispor.tefor.net). *COPB1* gRNAs: 5'-CAGGTTATCAAAGCGCTGAA-3' and 5'-AGGTAGCACAAAACGAATGA-3'. *RAB5* gRNAs: 5'-CGAGGCGCAACAAGACCCAA-3' and 5'-GAGGCGCAACAAGACCCAAC-3'. The HIV-Gag-Pol packaging plasmid (psPAX2, Addgene plasmid# 12260; a gift from Dr. Didier Trono) was used to generate lentiviral particles harboring Cas9 and these gRNAs.

(iii) VSV-G construct. The plasmid for the expression Vesicular Stomatitis Virus (VSV) glycoprotein was obtained through Addgene (Addgene plasmid# 12259).

(iv) Plasmids coding for autophagy proteins. Human LC3B was cloned into the expression vector pQCXIP with an EGFP-tag attached to its N-terminus. pMX-EGFP-FYVE1, a gift from Dr. Noboru Mizushima (Addgene plasmid # 38269)(133) was used to express DFCP1/FYVE1. MLV Gag-Pol, a gift from Dr. Patrick Salmon (Addgene plasmid # 35614) was used to generate retroviral particles encoding for EGFP-LC3B.

The RAB5-RFP plasmid, a gift from Ari Helenius (Addgene plasmid # 14437)(135), was used to reconstitute *RAB5*KO cells.

(vi) ESCRT machinery dominant-negative construct. The plasmid encoding for the dominant-negative mutant of VPS4, $VPS4_{E228Q}$-EGFP, was a gift from Dr. Wesley Sundquist (Addgene plasmid # 80351)(134).

Cell culture.
Human HEK293T cells (American Type Culture Collection [ATCC], CRL-11268), HEK293T-ACE2 cells (Biodefense and Emerging Infections [BEI], NR-52511), HEK293T-ACE2-30F-PLP2 (National Institute for Biological Standards and Control [NIBSC], 101062), African green monkey VeroE6 cells (ATCC, CRL-1586), and human MRC5-ACE2 fibroblasts (a gift from Dr. Joshua C. Munger [University of Rochester Medical Center, Rochester, NY]), were cultured in complete medium (Dulbecco's Modified Eagle Medium [DMEM, ThermoFisher Scientific, 11885-084] supplemented with 10% fetal bovine serum [FBS, ThermoFisher Scientific, 26140-079], 1% Penicillin-Streptomycin [ThermoFisher Scientific, 15070-063] and 1% L-glutamine [ThermoFisher Scientific, 25030-081]). Human lung A549-ACE2 cells (BEI, NR-53821) were maintained in complete medium supplemented with 50 ng/mL blasticidin S HCl (ThermoFisher Scientific, A1113903). Human bronchial epithelial cells 16HBE (Lonza-CC-2540S) were cultured in complete medium supplemented with 1x Non-Essential Amino Acids (ThermoFisher Scientific, 11140050), 100 nM Sodium Pyruvate (ThermoFisher Scientific, 11360070), and 7.5 mL of 1 M HEPES Buffer (ThermoFisher Scientific, 15630080).

VeroE6 cells stably expressing EGFP-LC3, VeroE6 *ATG5*KO, VeroE6 *RAB5*KO, MRC5-ACE2 *ATG5*KO, MRC5-ACE2 *RAB5*KO, HEK293T-ACE2 *ATG5*KO, HEK293T-ACE2 *RAB5*KO, and HEK293T-ACE2-30F-PLP2 *RAB5*KO cells were cultured in complete medium supplemented with 1 ng/mL of puromycin. A549-ACE2 *ATG5*KO or *RAB5*KO cells were cultured in complete medium supplemented with 50 ng/mL blasticidin S HCl and 2 ng/mL of puromycin. 16HBE *COPB1*KO cells were cultured in complete medium

supplemented with 1x Non-Essential Amino Acids, 100 nM Sodium Pyruvate, 7.5 mL of 1 M HEPES Buffer and 1 ng/mL of puromycin.

Transfections.

HEK293T and HEK293T-ACE2 cells were transfected using GenJet *in vitro* DNA transfection reagent (SignaGen Laboratories, SL100488), following the manufacturer's instructions, including total DNA, dish size, incubation time, and the ratio of GenJet: DNA for optimal transfection efficiency. VeroE6 cells were transfected using Lipofectamine 3000 Transfection Reagent (ThermoFisher Scientific, L3000001) following the manufacturer's instructions. Cell viability was monitored for every transfection to evaluate if the expression of the transgene could cause cellular toxicity. No evidence of toxicity was observed. If viability was below 85%, cells were considered unsuitable for further analyses. Viabilities were usually above 90%.

Generation of VeroE6 EGFP-LC3 stably expressing cells.

(i) VLP generation. 5 x 10^6 HEK293T cells were seeded in 25 cm^2 flasks. Twenty-four h later, cells were transfected with 3.45 μg MLV-Gag-Pol packaging plasmid, 1.725 μg VSV-G envelope expressing plasmid, and 6.9 μg EGFP-LC3B plasmid. The supernatant was collected 48 h post-transfection, centrifuged for 10 min at 931 *x g* to remove the cell debris, aliquoted into 1 mL cryotubes, and stored at -80ºC.

(ii) Transduction. 5 x 10^6 VeroE6 cells were seeded in 25 cm^2 flasks. Twenty-four h later, cells were transduced with VLPs harboring EGFP-LC3B. Forty-eight h later, the cell medium was replaced and supplemented with 1 ng/mL of puromycin. Cells were cultured under puromycin for 10 days to allow for the selection of cells successfully transduced with EGFP-LC3B. The expression level of EGFP-LC3B was verified by fluorescence microscopy and flow cytometry.

Generation of knockout cells using CRISPR-Cas9 genome editing.

(i) VLP generation. 5 x 10^6 HEK293T cells were seeded in 25 cm^2 flasks. Twenty-four h later, cells were transfected with 3.45 μg HIV-Gag-Pol packaging plasmid, 1.725 μg

VSV-G envelope expressing plasmid, and 6.9 μg LentiCRISPRv2-ATG5/RAB5/COPB1 plasmids. The supernatant was collected 48 h post-transfection and centrifuged for 10 min at 931 *x g* to remove the cell debris, aliquoted into 1 mL cryotubes, and stored at -80ºC.

(ii) Transduction. 5 x 10^6 VeroE6, A549-ACE2, MRC5-ACE2, HEK293T-ACE2, 16HBE, and HEK293T-ACE2-30F-PLP2 cells were seeded in 25 cm^2 flasks. Twenty-four h later, cells were transduced with VLPs harboring Cas9 and the gRNA targeting *ATG5*, *RAB5* or *COPB1*. Forty-eight h later, the cell medium was replaced and supplemented with puromycin. Cells were cultured under puromycin for 10 days to allow for the selection of cells successfully transduced with Cas9 and the corresponding gRNAs. Cells were then harvested for knockout assessment by western blotting and phenotypic analyses.

Knockdown assays.

esiRNA oligos were obtained from ThermoFisher Scientific to deplete human *AP1*, *AP2*, and *COPB1* (138604, 14075, s3373, respectively). A control siRNA SignalSilence was used (ThermoFisher Scientific, 6568). Knockdown of target genes was achieved by transient transfection of the esiRNAs in 10^6 HEK293T-ACE2 cells using Lipofectamine 3000 *in vitro* transfection reagent, following the manufacturer's instructions (ThermoFisher Scientific, L3000015). Knockdown was verified by western blot.

Drug treatments.

(i) Autophagy-related drugs. VeroE6 cells were treated with 3-methyladenine (3-MA; Sigma-Aldrich, M9281, 0-10 mM), VPS34IN (Selleckchem, S7980, 0-25 μM), Torin2 (Selleckchem, S2817, 0-0.5 μM), Vistusertib (Selleckchem, S2783, 0-0.5 μM), Dactolisib (Selleckchem, S1009, 0-2.5 μM), and Wortmannin (Selleckchem, S2758, 0-0.02 mM) 2 h post SARS-CoV-2 HK infection (MOI = 1). The drug treatments were maintained for 4 h.

(ii) Endocytosis inhibitors. VeroE6, A549-ACE2, MRC5-ACE2, HEK293T-ACE2, and 16HBE cells were treated with pitstop2 (Abcam, 120687, 100 μM), or dynasore (Sigma-

Aldrich, D7693, 80 μM) 4.5 h or 8.5 h post-SARS-CoV-2 HK infection (MOI = 1). Treatments were maintained for 1.5 h.

(iii) Golgicide and Brefeldin A treatment. 16HBE cells were treated with 1-5 μM of Golgicide (Sigma-Aldrich, G0923) and Brefeldin A (Sigma-Aldrich, B5936) 2 h after infection with SARS-CoV-2 HK (MOI = 1). Treatments were maintained for 8 h.

(iv) K22 treatment. VeroE6, A549-ACE2, MRC5-ACE2, HEK293T-ACE2, and 16HBE cells were infected with SARS-CoV-2 HK (MOI = 1). 2 h later, cells were treated with increasing concentrations of (0-80 μM) of K22 (Caymanchem, 31578) for 4 h (8 h for 16HBE cells).

XTT assay.

To measure changes in cell viability due to drug treatments, the colorimetric assay XTT (2,3-bis-[2-methoxy-4-nitro-5-sulfophenyl]-2H-tetrazolium-5-carboxanilide, Sigma-Aldrich, 11465015001) was used. For this, 2 x 10^4 VeroE6 cells were seeded in 96-well plates. Eighteen h later, cells were treated with pitstop 2 (100 μM). Six h later, XTT reagents were added to the cell medium, and incubated for 2 h before reading the assay. Absorbance measurements were then taken at 475 nm and 650 nm for each treatment condition using a microplate reader (BMG LABTECH, LUMIstar Omega). The metabolic readout was calculated by subtracting the absorbance measured at 650 nm from that obtained at 465 nm. Measurements were done in 3 technical replicates and experiments were repeated three independent times.

SARS-CoV-2 infections.

All SARS-CoV-2 infection experiments were performed at the URMC Biosafety Level 3 laboratory, following the approved standard operating procedures.

One million HEK293T-ACE2 cells or 5 x 10^5 VeroE6, A549-ACE2, MRC5-ACE2, and 16HBE cells were seeded in 6-well plates. Eighteen h later, cells were infected with SARS-CoV-2 isolate Hong Kong (BEI resources, NR52282) at MOI = 1, unless noted

differently, diluted in DMEM with 3% FBS. Infections were kept at 4ºC to synchronize the infection. Two h post-infection, virus inoculum was removed. Cells were then washed twice with DPBS (ThermoFisher Scientific, 14190144) and incubated with DMEM supplemented with 10% FBS. Two and/or 6 h post-infection (10 h for HBE cells), cells were lysed with 1 mL Trizol (for RNA extraction; ThermoFisher Scientific, 15596018) or 0.3 mL lysis IP buffer (ThermoFisher Scientific, 87787) supplemented with 1% Triton X-100. Culture supernatants were collected for virus titration. For SARS-CoV-2 kinetics assays, RNA was extracted at 2-, 4-, 6-, 8-, and 10-h post-infection. Similarly, supernatants were collected at those times to evaluate virion production (see $TCID_{50}$ section below). Similar infections were performed in the presence of siRNAs, cells depleted from ATG5, RAB5, or COPB1, or in the presence of drugs targeting autophagy, endocytosis, Golgi transport, or NSP6.

Luciferase reporter assay.

One million parental and *RAB5*KO HEK293T-ACE2-30F-PLP2 cells were infected with SARS-CoV-2 HK at MOIs 2 and 5. Four h later, cells were washed and harvested to measure firefly and renilla luciferase activities by luminescence using a microplate reader (BMG LABTECH, LUMIstar Omega). As controls, uninfected cells were included for both parental and *RAB5*KO cells, which were used to subtract any background firefly luminescence. Data is presented as firefly luciferase over renilla luciferase.

RT-qPCR.

(i) RNA extraction and cDNA synthesis. SARS-CoV-2 infected cells were washed with DPBS (ThermoFisher Scientific, 14190144) and total RNA was extracted by adding 1 mL Trizol (ThermoFisher Scientific, 15596018) per well. Next, 200 μL of chloroform (Spectrum, C1220) were added, and samples were centrifuged at 12,000 *x g* for 15 min at 4ºC, which created three distinct density phases: a lower red phenol-chloroform phase, an interphase, and an RNA-containing transparent aqueous phase at the top. The RNA phase was collected and mixed with 500 μL of isopropyl alcohol to precipitate RNA. RNA was then washed with 75% ethanol and eluted in RNase free water (ThermoFisher Scientific, 10977015). Finally, 1 μg of purified RNA was reverse

transcribed and converted into cDNA using the iScript cDNA synthesis kit (Bio-Rad, 1725037), following the manufacturer's instructions. cDNA samples were used for qPCR analysis.

(ii) qPCR. To measure the levels of SARS-CoV-2 RNA, the SYBR green-based real-time qPCR method was used. Primer pairs specific for portions of the SARS-CoV-2 *ORF1ab*, a reading frame exclusively present in genomic RNA, were designed. Specifically, primers targeting *NSP12* (FW: AATAGAGCTCGCACCGTAGC, RV: CATGTTGTGCCAACCACCAT) and *NSP6* (FW: ATGGTGCTAGGAGAGTGTGG, RV: AGAGCCCACATGGAAATGGC). For quality control, the following qPCR primers were included to examine for RNA integrity (PrimePCR RNA assay RQ1 and RQ2 primers, Bio-Rad, 10025694), genomic DNA contamination (PrimePCR gDNA, Bio-Rad, qHsaCtID0001004), and housekeeping gene levels (PrimePCR human *GAPDH*, Bio-Rad, qHsaCED0038674; PrimePCR human *ACTB*, BioRad qHsaCEP0036280; and PrimePCR human *CALR*, BioRad qHsaCIP0030886). Each PCR reaction consisted of 5 μL 2x SsoAdvanced universal SYBR green supermix (Bio-Rad, 1725272), 0.1 μL cDNA, 4.4 μL RNase free water (ThermoFisher Scientific, 10977015), and 0.5 μL qPCR primer pairs (*NSP12*, *NSP6* or control primers). The amplification program was as follows: 2 min at 95°C for initial activation, 40 cycles at 95°C for 5 seconds, 60°C for 30 seconds, and then melting analyses from 65 to 95°C (0.5°C increments). Each sample was analyzed by qPCR in two technical replicates. Experiments were performed three independent times for each experimental condition. Changes in *NSP12* and *NSP6* RNA levels was determined by the $2^{\Delta\Delta Cq}$ method using *GAPDH* as the housekeeping gene, which allowed to obtain the relative levels of SARS-CoV-2 genomic RNA for each time point and treatment condition. SARS-CoV-2 genome replication was calculated as fold-change over basal viral RNA. A fold change of >2.0 or <0.5 relative to the control samples was considered biologically relevant (58).

Median Tissue Culture Infectious Dose Assay ($TCID_{50}$).

To titrate SARS-CoV-2 virus stocks and culture supernatants from infections, virus samples were serially diluted in DMEM with 3% FBS. 2 x 10^4 VeroE6 cells were seeded

in 96-well plates. Twenty-four h later, culture media were removed from the plates, and cells were infected with 100 μL of each virus dilution. Each dilution was tested in 6 replicates. Three days post-infection, the cytopathic effect (CPE) was determined by optical microscopy and the $TCID_{50}$ value was calculated using the Spearman-Kärber method (139).

Western blotting.

Transfected cells were washed with DPBS (ThermoFisher Scientific, 14190144) and harvested in 0.3 mL lysis IP buffer (ThermoFisher Scientific, 87787) complemented with protease inhibitors (Roche, 04693116001) and phosphatase inhibitor cocktails 2 and 3 (Sigma-Aldrich, P5726 and P0044). SARS-CoV-2-infected cells were washed the same way, harvested in lysis IP buffer (ThermoFisher Scientific, 87787) complemented with protease inhibitors (Roche, 04693116001), phosphatase inhibitor cocktails 2 and 3 (Sigma-Aldrich, P5726 and P0044) and 1%Triton X-100 (Sigma-Aldrich, X100). Cell lysates were then incubated on ice for 30 min. Samples were centrifuged at 16,000 x *g* at 4°C for 8 min to remove cell debris. Supernatants were collected, mixed with 2x SDS sample buffer (Sigma-Aldrich, S3401), and boiled for 10 min on a heat block. Proteins were then separated by electrophoresis on 12% SDS-PAGE polyacrylamide gels and transferred to a polyvinylidene difluoride (PVDF) membrane (BioRad, 1620177) using a Trans-Blot Turbo Transfer System (Bio-Rad, 1704150EDU). Membranes were then incubated for 1 h with blocking buffer (Bio-Rad, 1706404) at room temperature, followed by an overnight incubation at 4ºC with a primary antibody against our protein of interest (antibody sources and dilutions are detailed in **Table 1**). The following day, membranes were washed with PBS-tween (Sigma-Aldrich, P3563) 3 times for 15 min at room temperature, followed by a 60-min incubation with the corresponding secondary antibody (antibody sources and dilutions are detailed in **Table 1**) at room temperature. Next, the membranes were washed 3 additional times in PBS-tween (Sigma-Aldrich, P3563). Finally, membranes were developed with SuperSignal West Femto maximum sensitivity substrate (ThermoFisher Scientific, 34095), and visualized in a ChemiDoc MP imaging system (Bio-Rad, 12003154). The expression level of proteins was quantified using Image Lab software (Bio-Rad, Hercules, CA).

Immunoprecipitation assays.

One million HEK293T cells were transfected with 500 ng of a construct coding for SARS-CoV-2 *NSP3,* 500 ng of a plasmid coding for SARS-CoV-2 NSP4, and either 1,500 ng of a construct encoding SARS-CoV-2 *NSP6*-HA or 1,500 ng of an empty vector. Similar transfections were performed to assess the association between NSP6 and NSP12 (RdRp). In this case, cells were also transfected with 1,500 ng of NSP12 plasmid. Of note, NSP3 and NSP4 were included in these transfections to ensure proper expression and subcellular distribution of NSP6. Forty-eight h post-transfection, cells were washed with DPBS and incubated with membrane lysis buffer (Sigma-Aldrich, 4719956001) on ice for 30 min. The whole cell lysates were then incubated for 1 h at room temperature with Protein G magnetic beads (New England Biolabs, S1430S) to remove proteins unspecifically bound to the beads. In parallel, fresh Protein G magnetic beads were coated with an antibody against HA, RAB5 or COPB1 (**Table 1**) for 1 h at room temperature. Next, pre-cleared lysates were incubated with the antibody-coated Protein G beads overnight at 4°C. The next day, the beads were washed with lysis IP buffer (ThermoFisher Scientific, 87787) 5 times and resuspended in 2x SDS sample buffer (Sigma-Aldrich, S3401). Samples were analyzed by western blotting. As controls, cell lysates incubated with beads (but not coated with antibody; beads control) as well as a sample consisting of IP lysis buffer mixed with beads and antibody (IgG control) were included. These controls helped rule out any unspecific bands detected by western blot that corresponded to the IgG heavy or light chains or material from the magnetic beads.

Endosomal fractionation assay.

One million HEK293T cells were seeded in 6-well plates. Twenty-four h later, cells were transfected with 400 ng of a plasmid encoding SARS-CoV-2 NSP3, 400 ng of a construct encoding SARS-CoV-2 NSP4, 1,300 ng of the SARS-CoV-2 NSP6-HA plasmid and 400 ng of an expression vector coding for streptavidin-tagged RdRp (NSP12). As a control, cells were transfected with 2,500 ng of a plasmid coding for GST-HA. Forty-eight h post-transfection, cells were harvested, washed once on ice-cold

DPBS, and lysed to obtain early endosomal and cytosolic fractions using the Trident Endosome Isolation kit (GenTex, GTX35192), following the manufacturer’s instructions. Whole cell lysate, cytosolic and early endosomal fractions were analyzed by western blot for the presence of NSP6 (HA), RdRp (Strep) and the GST control (HA). Purity of the fractions was confirmed using antibodies against specific early endosomal markers (RAB5, EEA1), late endosomal markers (RAB7), cytosolic markers (non-prenylated RAB5), plasma membrane markers (Na/K ATPase), and cytoskeleton markers (ACTB). Partial presence of RAB7 in the early endosomal fraction is expected, as the transition from early to late endosomes requires a RAB cascade where RAB5 and RAB7 temporarily co-exist (140-142).

Fluorescence microscopy.

(i) SARS-CoV-2 RTC and LC3-GFP distribution. 5 x 10^4 VeroE6 EGFP-LC3B stably expressing cells were seeded in sterile tissue culture-treated 8-well slides. Eighteen h later, cells were infected with SARS-CoV-2 HK at MOI = 1. Six h post-infection, cells were washed with DPBS (ThermoFisher Scientific, 14190144), permeabilized, and fixed by incubating in 1:1 acetone-methanol (Sigma-Aldrich, 270725, 34860) at room temperature for 1 h. Cells were then blocked with antibody diluent solution (2% fish skin gelatin [Sigma-Aldrich, 67765] + 0.1% Triton X-100 [Sigma-Aldrich, X100] + 10% goat serum [ThermoFisher Scientific, 500062Z] with 1 x DPBS) for 30 min at room temperature and incubated for 60 min with the following primary antibody cocktail: antibody diluent solution + mouse monoclonal anti-dsRNA at a dilution of 1:200 (**Table 1**), or rabbit polyclonal anti-SARS-CoV-2 NSP12 at a dilution of 1:100 (**Table 1**). Subsequently, cells were washed with wash buffer (2% fish skin gelatin + 0.1% Triton X-100 with 1 x DPBS) 3 times and incubated with a goat anti-mouse IgG_{2a} secondary antibody conjugated with an Alexa 568 fluorophore, or a goat polyclonal anti-rabbit IgG antibody conjugated with Alexa 568 at a dilution 1:500 for 30 min (**Table 1**). Next, cells were washed with wash buffer 3 times and incubated with Hoechst (ThermoFisher Scientific, H3570; 1:5,000 dilution) for 3 min to visualize cell nuclei. After staining, the slides were washed and mounted using an anti-quenching mounting medium (Vector Laboratories, 3304770). The slides were visualized in a BioTek Lionheart FX automated

microscope and a Nikon A1R HD with TIRF confocal microscope using 40x and 60x/1.49 oil objectives, respectively, and filter cubes/excitation diodes 350 nm, 488 nm, and 586 nm in order to excite DAPI, EGFP, and TexasRed, respectively. Images were processed and analyzed using the Gen5 software (BioTek Instruments, Winooski, VT). Proportional adjustments of brightness/contrast were applied.

Analyses for the distribution of the SARS-CoV-2 RTC relative to other autophagy markers (FYVE1/DFCP1: an early phagophore marker; WIPI2: omegasome marker; SQSTM1/p62: an autophagy receptor marker; EDEM1: an EDEMosome marker; or endogenous LC3 [see antibodies in **Table 1**]) were performed in VeroE6 cells, following a similar procedure as the one detailed above. For DFCP1, VeroE6 cells were transfected with 0.5 μg of pMX-EGFP-FYVE1/DFCP1, which encodes for an EGFP-DFCP1 fusion construct. In this case, cells were infected with SARS-CoV-2 and processed in a similar manner as in the EGFP-LC3B assays. A549-ACE2, HEK293T-ACE2, and MRC5-ACE2 cells infected with SARS-CoV-2 were also analyzed for endogenous LC3 in a similar manner as VeroE6 cells.

(ii) SARS-CoV-2 RTC distribution relative to intracellular membranes. The subcellular distribution of the SARS-CoV-2 RTC relative to intracellular membrane markers (RAB5: early endosomes; CD63: late endosomes; RAB11: recycling endosomes; GOSR1: *cis*-Golgi; TGN46: *trans*-Golgi; CD81: exosomes; Calnexin: ER; ADRP: lipid droplets; and Mito-tracker [Thermo Scientific, C10593]: mitochondria) was investigated in SARS-CoV-2-infected VeroE6 cells using the antibodies shown in Table 1. A549-ACE2, HEK293T-ACE2, and MRC5-ACE2 infected with SARS-CoV-2 were also analyzed for the distribution of the virus RTC relative to RAB5 in a similar manner.

(iii) NSP6 distribution relative to RAB5, COPB1 and dsRNA. The subcellular distribution of SARS-CoV-2 NSP6 relative to RAB5, COPB1 and the virus dsRNA was investigated in SARS-CoV-2-infected VeroE6 cells or VeroE6 cells transfected with SARS-CoV-2 NSP3, NSP4 and NSP6 using the antibodies shown in Table 1 and similar procedures to the ones described above.

Co-localization between the cellular markers detailed above and SARS-CoV-2 NSP6, dsRNA or RdRp (NSP12) was evaluated by calculating the Pearson's correlation coefficient of 8 independent fields using ImageJ (NIH https://imagej.nih.gov/ij/).

Electron microscopy.

5 x 10^5 parental and *RAB5*KO VeroE6 cells were seeded in 6-well plates. Twenty-four h later, parental and *RAB5*KO cells were infected with SARS-CoV-2 HK at a MOI = 5. As a control, a set of uninfected, parental and *RAB5*KO cells were included. Six hours later, cells were fixed in 2.5% glutaraldehyde and 4% paraformaldehyde in 0.1 M sodium cacodylate buffer and incubated for 24 h at 4ºC. Next, cells were rinsed twice in buffer, post-fixed 45 min in 1.0% osmium tetroxide/1.5% potassium ferrocyanide in 0.1 M sodium cacodylate buffer. Afterwards, samples were washed twice in distilled water prior to entrapment in 3.0% agarose. The trapped pelleted cells were dehydrated in a graded series (20 min each) of ethanol to 100% (x 3 changes) and then transferred to 1:1 100% ethanol/propylene oxide (30 min), 100% propylene oxide (x 2 for 30 min each), 1:1 propylene oxide/Epon Araldite resin (2.5 hours) and finally 100% Epon Araldite resin overnight. The next day, the samples were embedded into fresh resin in silicone molds and polymerized for 48 h at 65ºC. One-micron sections were cut and placed onto glass slides and stained with Toluidine blue to identify cells for thin sectioning at 70 nm using a diamond knife and a Leica ultramicrotome. The thin sections were placed onto formvar/carbon copper slot grids and stained with aqueous uranyl acetate and 0.3% lead citrate. The grids were imaged using a Hitachi 7650 transmission electron microscope and an AMT 12 MP NanoSprint12 digital camera.

Statistical analysis.

Statistical calculations for 2-group comparisons were performed with a two-tailed unpaired Student T test. All other statistical comparisons were performed with One-Way ANOVA with Dunnet post hoc testing. Analyses were performed using Graph Pad Prism version 8.3.0. P values ≤ 0.05 were considered statistically significant.

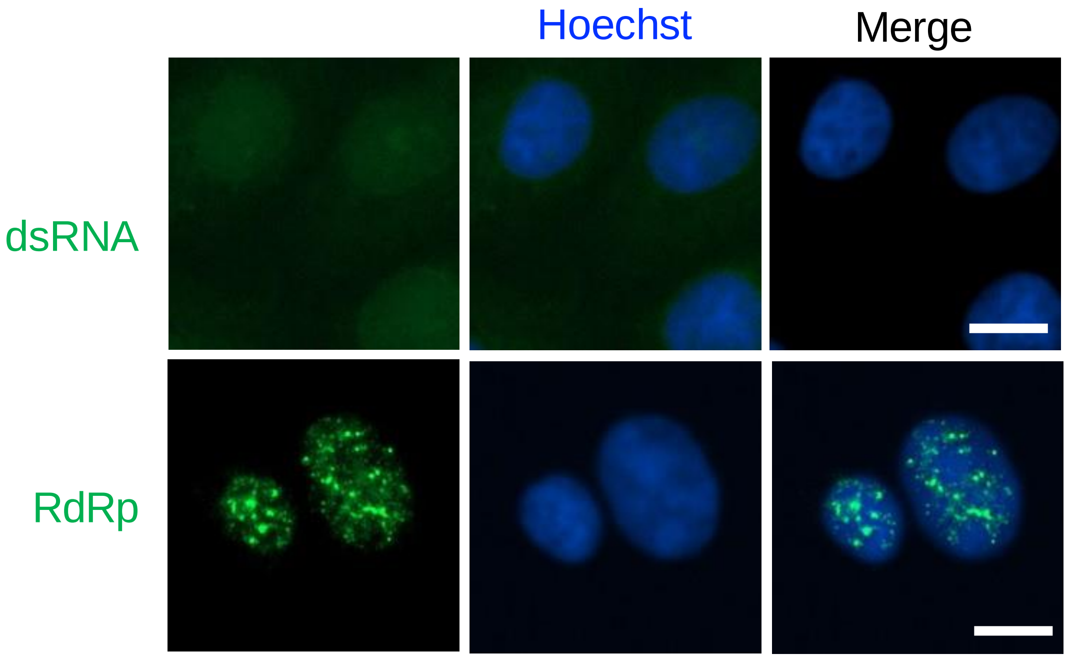
Hoechst
Merge
dsRNA
RdRp

SUPPLEMENTARY FIGURES

FIG S1. Staining for SARS-CoV-2 dsRNA is specific for infected cells, while staining for the virus RdRp cross-reacts with nuclear factors. Representative images of uninfected VeroE6 cells stained for dsRNA and SARS-CoV-2 NSP12. White bar: 10 μm.

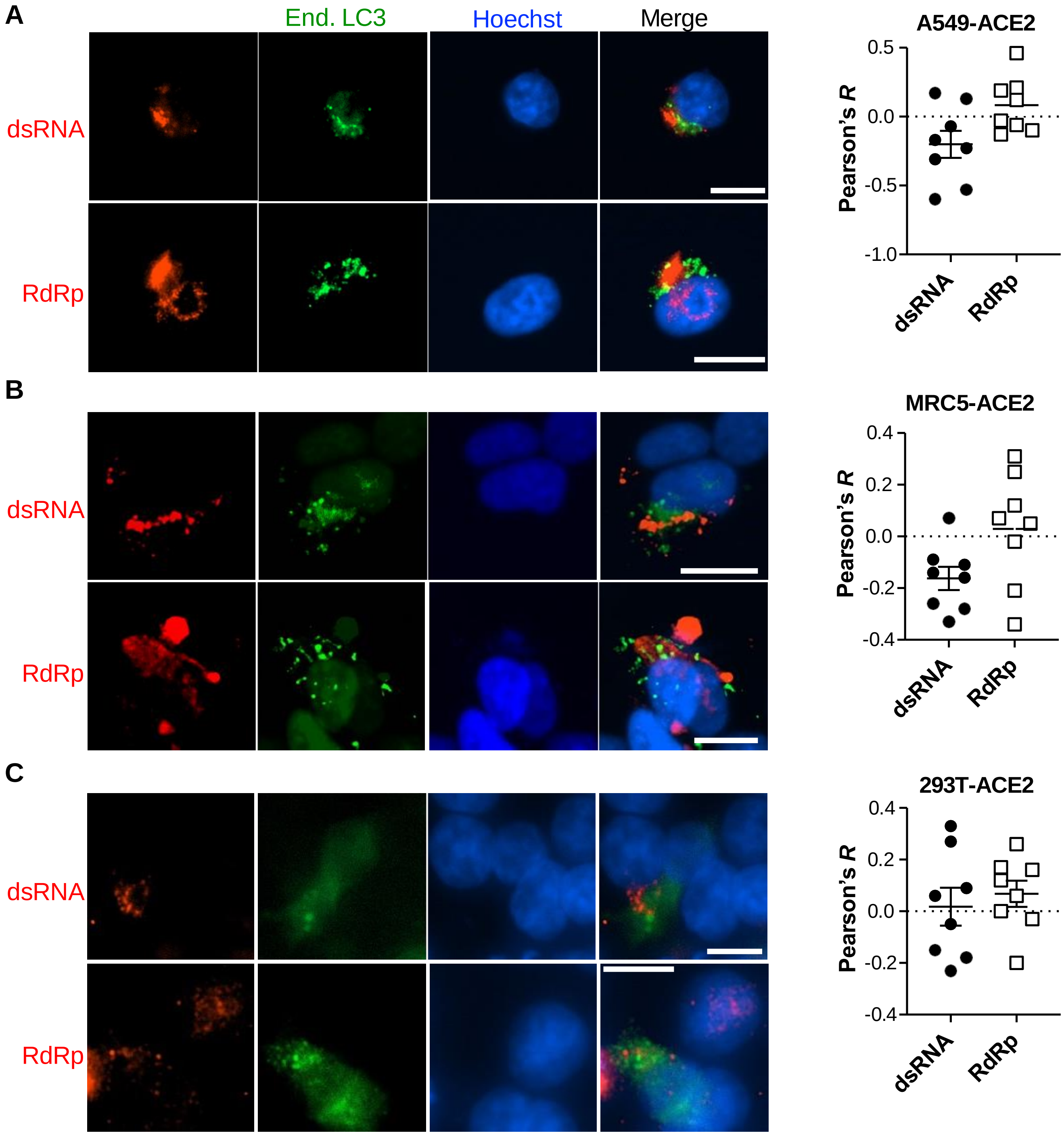
A
End. LC3
Hoechst
Merge
dsRNA
RdRp
A549-ACE2
Pearson's R
0.5
0.0
-0.5
-1.0
dsRNA
RdRp
B
dsRNA
RdRp
MRC5-ACE2
Pearson's R
0.4
0.2
0.0
-0.2
-0.4
dsRNA
RdRp
C
dsRNA
RdRp
293T-ACE2
Pearson's R
0.4
0.2
0.0
-0.2
-0.4
dsRNA
RdRp

FIG S2. SARS-CoV-2 dsRNA and RdRp are not found at LC3 locations. (**A**) A549-ACE2, (**B**) MRC5-ACE2, and (**C**) HEK293T-ACE2 cells were infected with SARS-CoV-2 HK (MOI = 1) in a synchronized manner. 6 h later, cells were fixed, blocked, and stained for endogenous LC3 (green) and the virus dsRNA or RdRp (red) to assess their degree of overlap. Pictures are representative of 3 independent experiments. Graphs: The Pearson's correlation coefficient (*R*) value for the co-localization of SARS-CoV-2 dsRNA (black circles) and RdRp (open squares) with endogenous LC3 was calculated from 8 randomly selected fields. White scale bar: 10 μm.

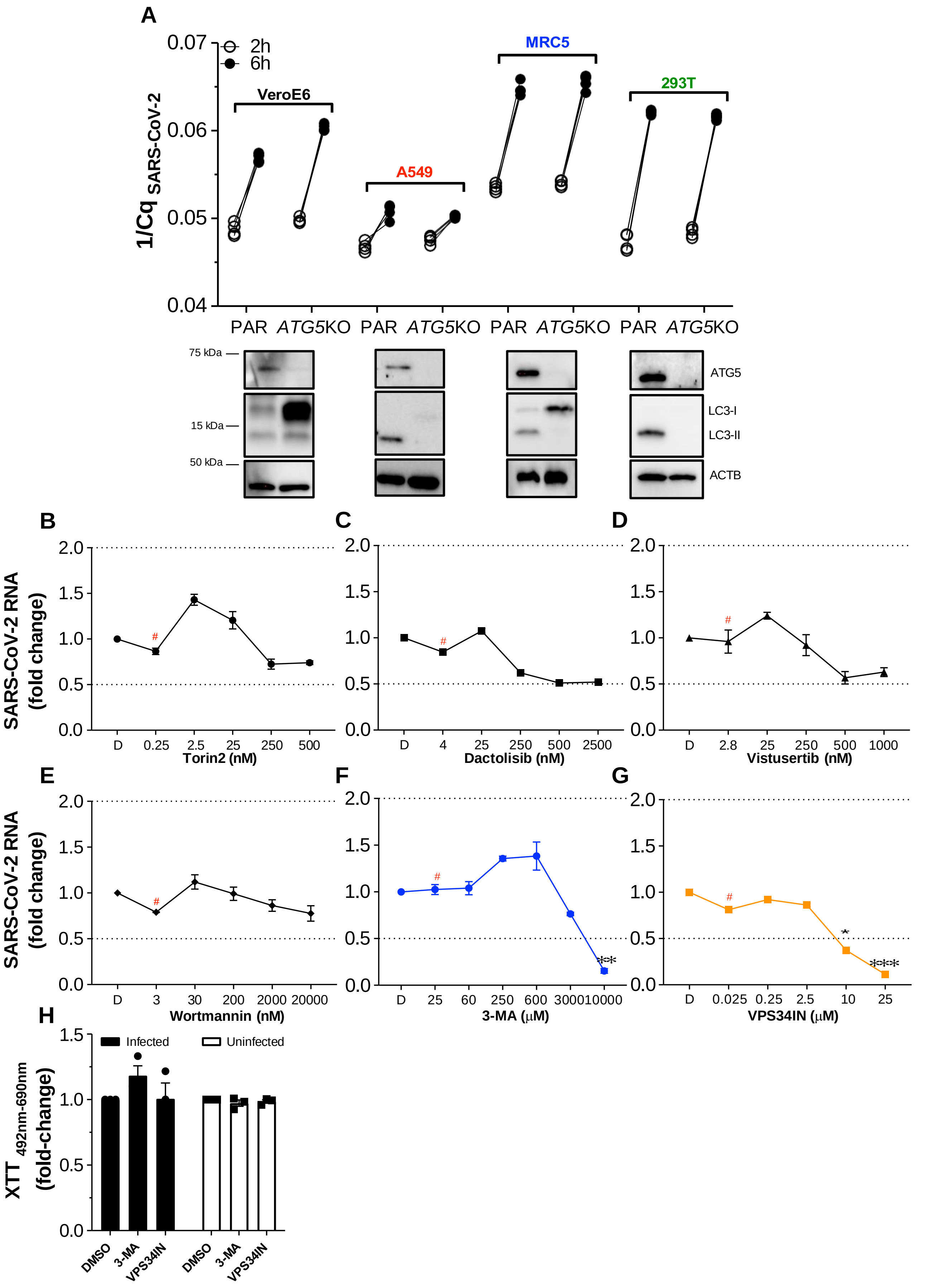

A
1/Cq SARS-CoV-2
2h
6h
VeroE6
A549
MRC5
293T
PAR
ATG5KO
75 kDa
15 kDa
50 kDa
ATG5
LC3-I
LC3-II
ACTB
B
C
D
E
F
G
H
SARS-CoV-2 RNA (fold change)
Torin2 (nM)
Dactolisib (nM)
Vistusertib (nM)
Wortmannin (nM)
3-MA (μM)
VPS34IN (μM)
XTT 492nm-690nm (fold-change)
Infected
Uninfected
DMSO
3-MA
VPS34IN

FIG S3. Functional autophagy is dispensable for SARS-CoV-2 RNA synthesis. (**A**) Parental and *ATG5*KO cells were infected with SARS-CoV-2 HK (MOI = 1) in a synchronized manner. Virus genome copy numbers were determined at 2 h and 6 h post-infection by RT-qPCR using primer pairs specific for *NSP12* and *NSP6* in the indicated parental and *ATG5*KO cells. Genome copies detected at these time points were expressed as the inverse Cq value (1/Cq) for each experimental replicate (4 total replicates). Bottom blots: representative western blots of parental and *ATG5*KO cells confirming ATG5 depletion and its effects on the lipidation of LC3. ACTB/β-actin was included as a loading control. (**B**-**G**) Differences in SARS-CoV-2 genome copy numbers were determined by RT-qPCR using primer pairs specific for *NSP12* and *NSP6* at the peak of genome replication in VeroE6 cells infected with SARS-CoV-2 HK (MOI = 1) in the presence of increasing concentrations of Torin2 (B), Dactolisib (C), Vistusertib (D), Wortmannin (E), 3-MA (F), or VPS34IN (G). The compounds were added 2 h post-synchronized infection. Cells were harvested 4 h later. SARS-CoV-2 RNA levels were normalized to DMSO (D on the x axis) and expressed as fold-change. (**H**) Effects of 3-MA at 10 mM and VPS34IN at 25 μM on cellular viability and toxicity were examined by XTT assays in both infected and uninfected cells. Data is expressed as fold-change over DMSO. *: $p<0.05$, **: $p<0.01$, ***: $p<0.001$. Red pound: IC_{50} for each inhibitor. Data correspond to the mean and SEM of 3 independent experiments.

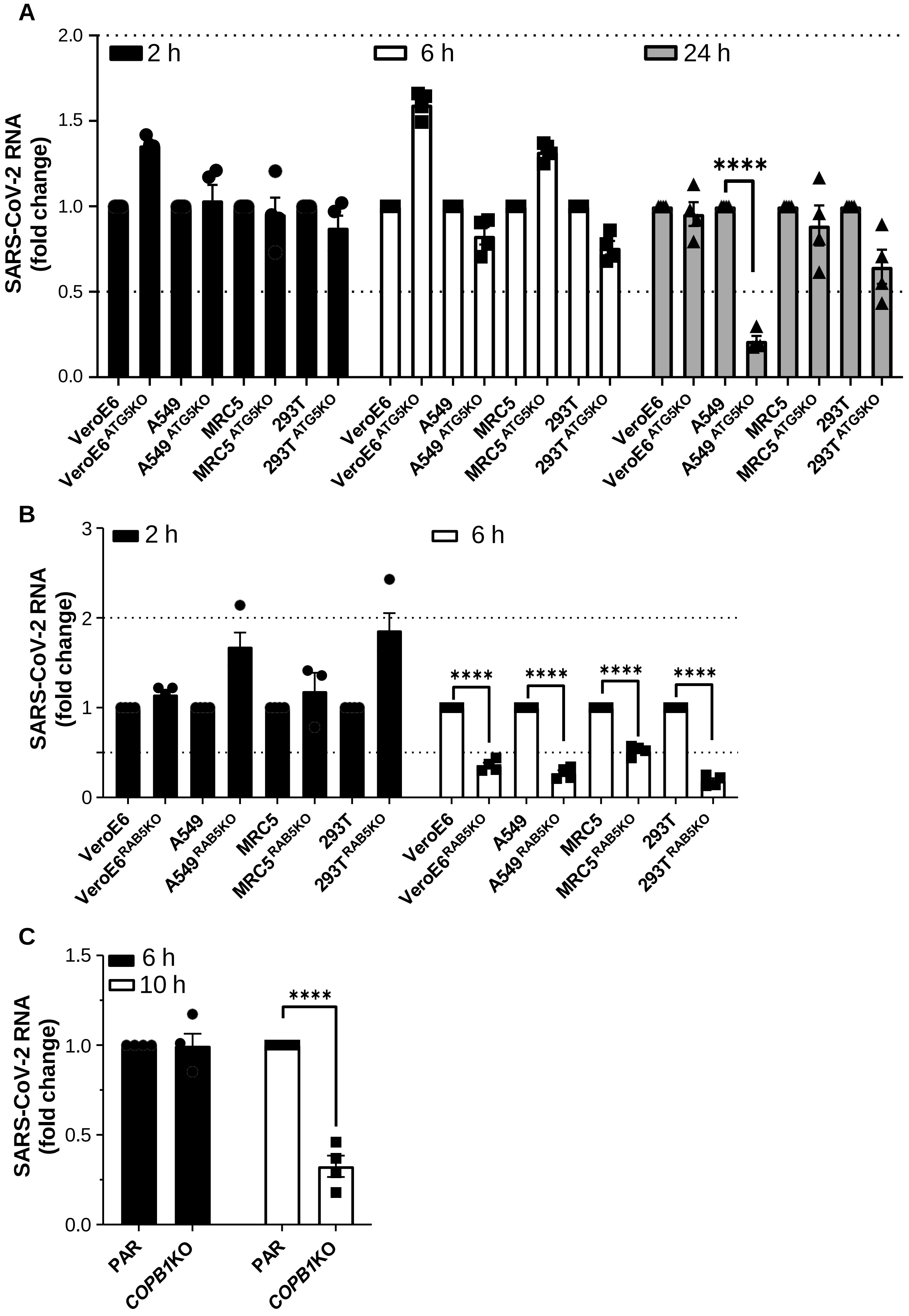
A
SARS-CoV-2 RNA (fold change)
2.0
1.5
1.0
0.5
0.0
2 h
6 h
24 h
****
VeroE6
VeroE6 ATG5KO
A549
A549 ATG5KO
MRC5
MRC5 ATG5KO
293T
293T ATG5KO
B
SARS-CoV-2 RNA (fold change)
3
2
1
0
2 h
6 h
****
VeroE6
VeroE6 RAB5KO
A549
A549 RAB5KO
MRC5
MRC5 RAB5KO
293T
293T RAB5KO
C
SARS-CoV-2 RNA (fold change)
1.5
1.0
0.5
0.0
6 h
10 h
****
PAR
COPB1KO

FIG S4. Effects of knocking out *ATG5*, *RAB5* and *COPB1* on SARS-CoV-2 entry. (**A**) Parental and *ATG5*KO cells, (**B**) Parental and *RAB*5KO cells and (**C**) Parental and *COPB1*KO 16HBE cells were infected with SARS-CoV-2 HK (MOI = 1) in a synchronized manner. Virus genome copy numbers were determined at 2 h, 6 h and 24 h post-infection by RT-qPCR using primer pairs specific for *NSP12* and *NSP6* and expressed as fold-change over parental cells for each time point. ****: $p<0.001$. Data correspond to the mean and SEM of 4 independent experiments.

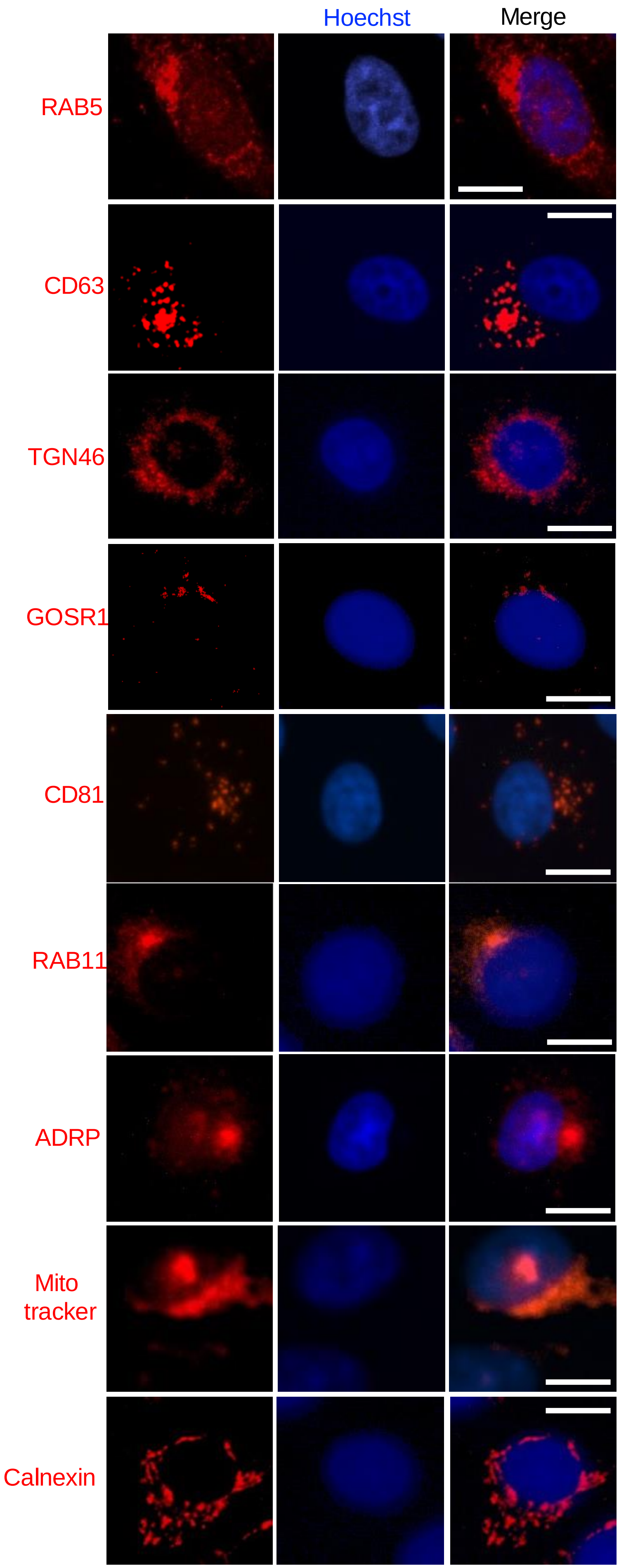
Hoechst
Merge
RAB5
CD63
TGN46
GOSR1
CD81
RAB11
ADRP
Mito tracker
Calnexin

FIG S5. Subcellular distribution of intracellular membrane markers in uninfected cells. To rule out any changes induced by SARS-CoV-2 in the expression and distribution of RAB5, CD63, TGN46, GOSR1, CD81, RAB11, ADRP, MitoTracker, and Calnexin – cellular markers used to measure their co-localization with the virus RTC – images were obtained from uninfected cells. Pictures are representative of 3 independent experiments. White scale bar: 10 μm.

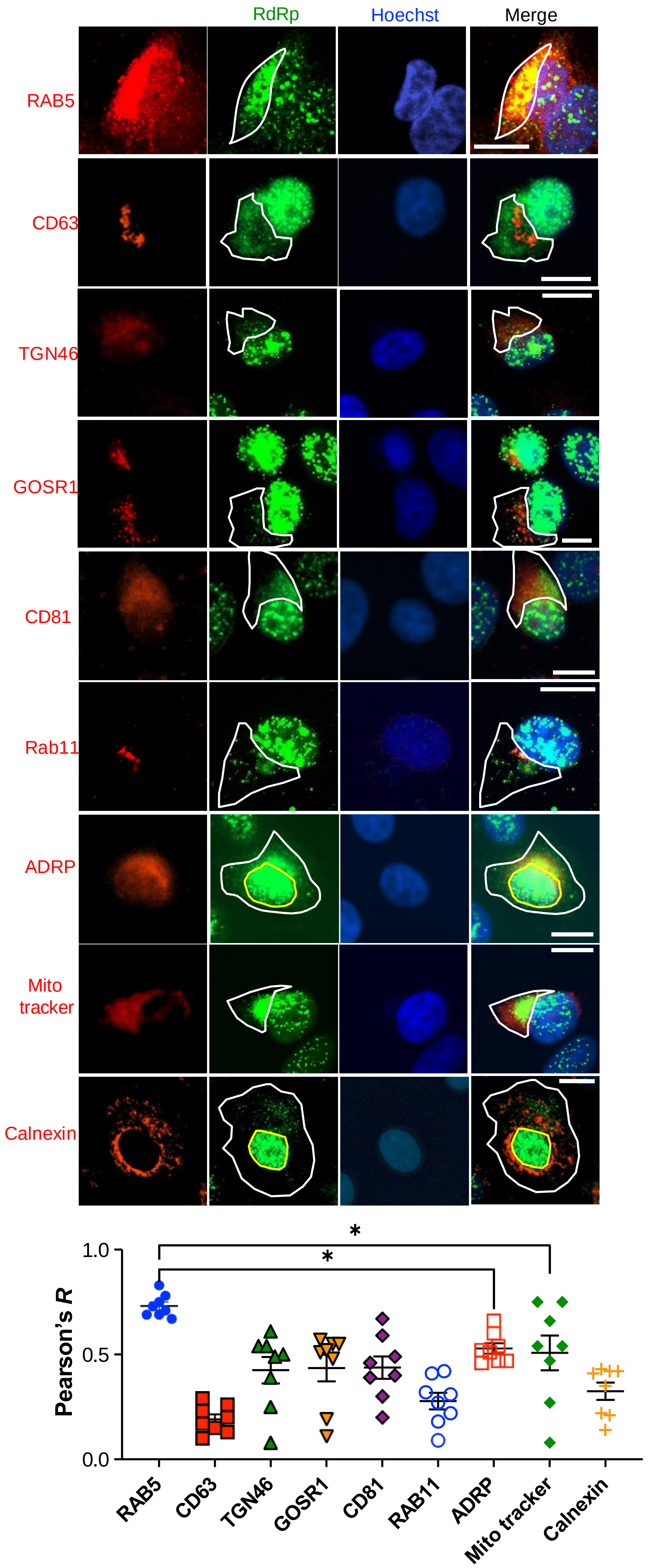

RdRp
Hoechst
Merge
RAB5
CD63
TGN46
GOSR1
CD81
Rab11
ADRP
Mito tracker
Calnexin
Pearson's R
1.0
0.5
0.0
*
*
RAB5
CD63
TGN46
GOSR1
CD81
RAB11
ADRP
Mito tracker
Calnexin

FIG S6. Early endosomal membranes highly overlap with the SARS-CoV-2 RdRp. VeroE6 cells were infected with SARS-CoV-2 HK (MOI = 1) in a synchronized manner. 6 h later, cells were fixed, blocked, and stained for the virus RdRp and the intracellular markers RAB5, CD63, TGN46, GOSR1, CD81, RAB11, ADRP, MitoTracker, and Calnexin. Images: representative pictures of 3 independent experiments. Graph: The Pearson's correlation coefficient (*R*) value for the co-localization of SARS-CoV-2 RdRp and the intracellular markers listed above was calculated from 8 randomly selected fields. Raw values with their mean and SEM are represented. White scale bar: 10 µm. Dotted white lines delineate the cytoplasm while yellow circles delineate the nucleus. *: $p< 0.05$

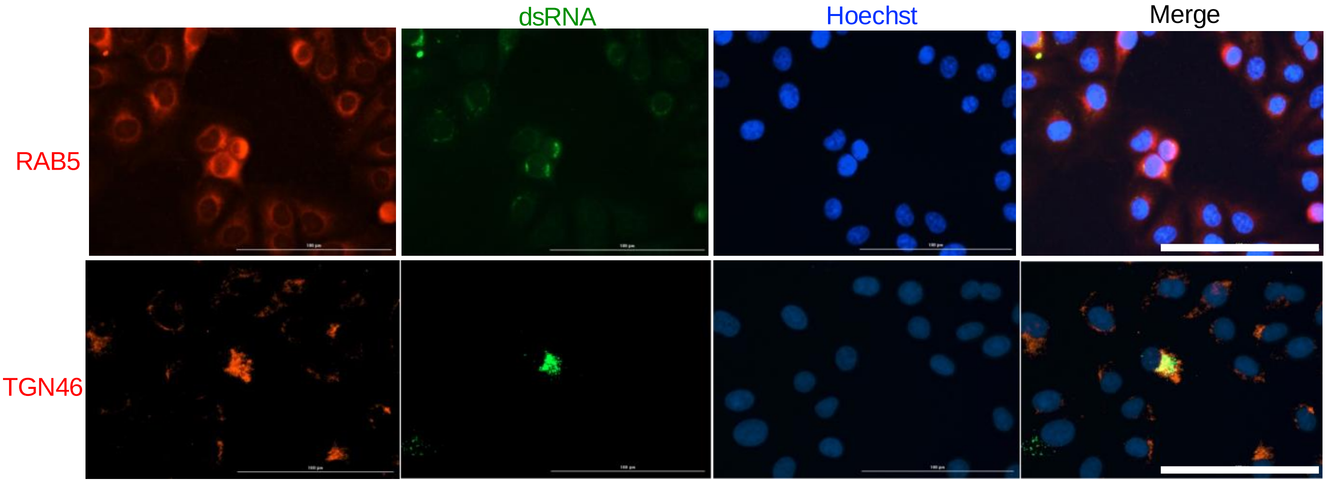

dsRNA
Hoechst
Merge
RAB5
TGN46

FIG S7. SARS-CoV-2 upregulates expression and distribution of endosomal and Golgi markers. VeroE6 cells were infected with SARS-CoV-2 HK (MOI = 1) in a synchronized manner. 6 h later, cells were stained for the virus dsRNA (green), the nuclei (blue) and either RAB5 or TGN46 (red). Images are representative of 3 independent experiments. White scale bar: 100 μm.

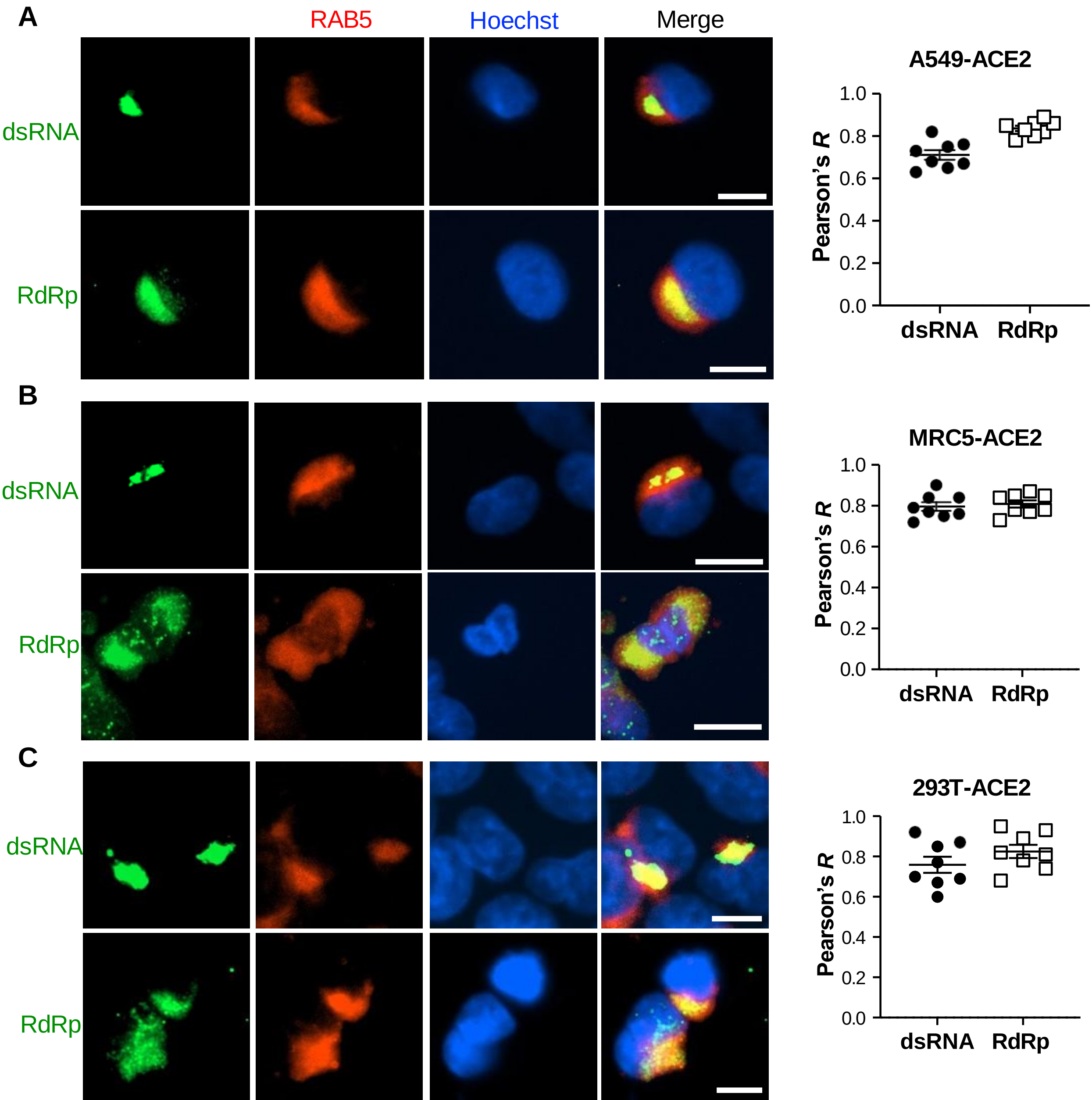

A
RAB5
Hoechst
Merge
dsRNA
RdRp
A549-ACE2
Pearson's R
1.0
0.8
0.6
0.4
0.2
0.0
dsRNA
RdRp
B
dsRNA
RdRp
MRC5-ACE2
Pearson's R
1.0
0.8
0.6
0.4
0.2
0.0
dsRNA
RdRp
C
dsRNA
RdRp
293T-ACE2
Pearson's R
1.0
0.8
0.6
0.4
0.2
0.0
dsRNA
RdRp

FIG S8. Early endosomal marker RAB5 overlaps with SARS-CoV-2 dsRNA and RdRp in human cells. (**A**) A549-ACE2, (**B**) MRC5-ACE2, and (**C**) HEK293T-ACE2 cells were infected with SARS-CoV-2 HK (MOI = 1) in a synchronized manner. 6 h later, cells were fixed, blocked, and stained for RAB5 and the virus RdRp or dsRNA. Images: representative pictures from 3 independent experiments. White scale bar: 10 μm. Graphs: The Pearson's correlation coefficient (*R*) value for the co-localization of RAB5 with the SARS-CoV-2 dsRNA (black circles) and RdRp (open squares) was calculated from 8 randomly selected fields.

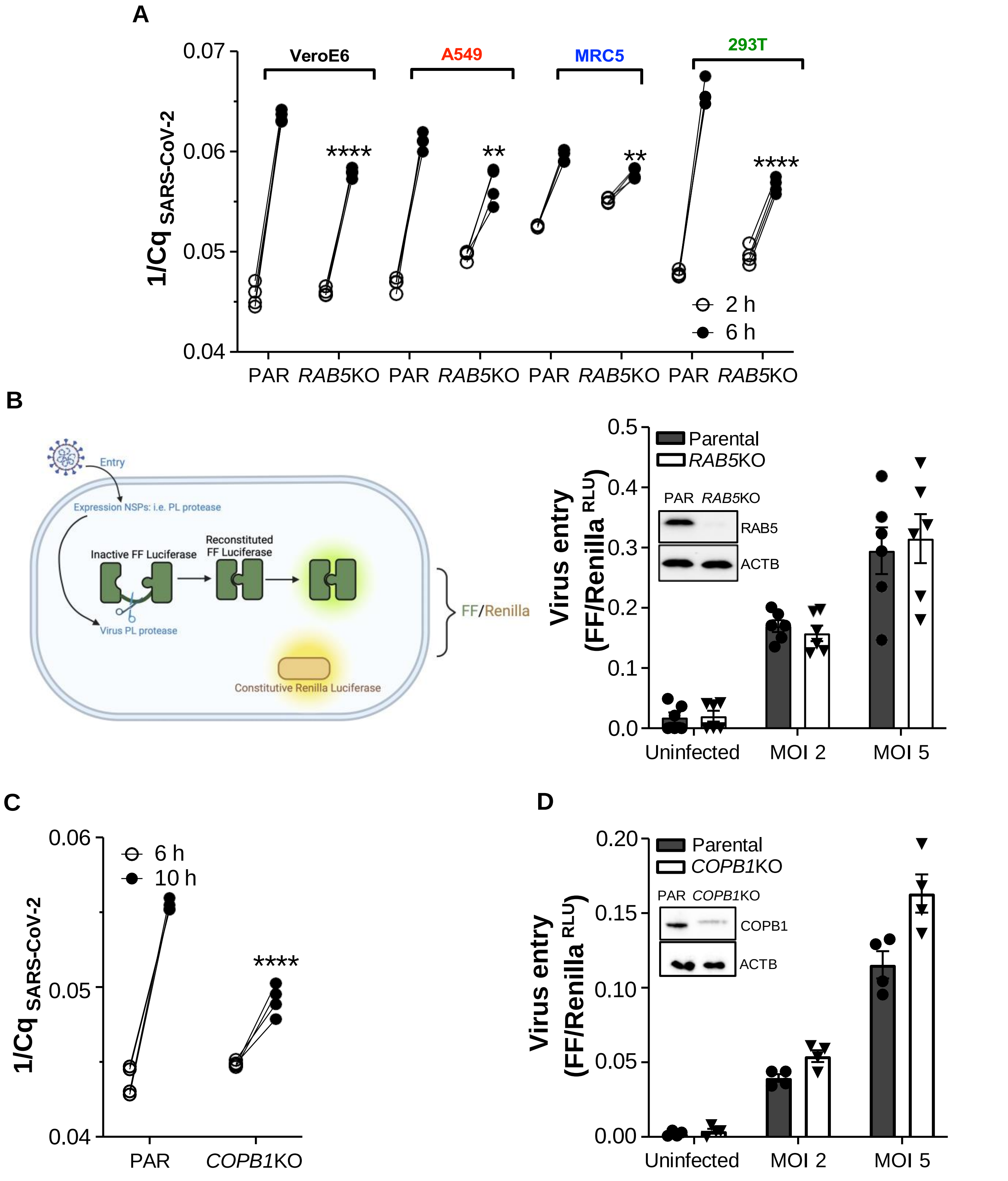
A
1/Cq SARS-CoV-2
0.07
0.06
0.05
0.04
VeroE6
A549
MRC5
293T
****
**
**
****
2 h
6 h
PAR RAB5KO PAR RAB5KO PAR RAB5KO PAR RAB5KO
B
Entry
Expression NSPs: i.e. PL protease
Inactive FF Luciferase
Reconstituted FF Luciferase
Virus PL protease
Constitutive Renilla Luciferase
FF/Renilla
Virus entry (FF/Renilla RLU)
0.5
0.4
0.3
0.2
0.1
0.0
Parental
RAB5KO
PAR RAB5KO
RAB5
ACTB
Uninfected
MOI 2
MOI 5
C
1/Cq SARS-CoV-2
0.06
0.05
0.04
6 h
10 h
****
PAR
COPB1KO
D
Virus entry (FF/Renilla RLU)
0.20
0.15
0.10
0.05
0.00
Parental
COPB1KO
PAR COPB1KO
COPB1
ACTB
Uninfected
MOI 2
MOI 5

FIG S9. Deletion of *RAB5* or *COPB1* has no negative impact on virus entry. (**A**) The indicated parental and *RAB5*KO cells were infected with SARS-CoV-2 HK (MOI = 1) in a synchronized manner. 2 h and 6 h post-infection, total RNA was extracted, and SARS-CoV-2 genome copy numbers were determined by RT-qPCR using primer pairs specific for *NSP12* and *NSP6*. SARS-CoV-2 RNA was expressed as the inverse of the raw Cq value (1/Cq) for each replicate and time point. (**B**) Cartoon: representation of the PL-activatable luciferase in HEK293T-ACE2-30F-PLP2 cells, created with *BioRender*. Graph: Parental and *RAB5*KO HEK293T-ACE2-30F-PLP2 cells were infected with SARS-CoV-2 HK (MOIs 2 and 5). 4 h later, virus entry was examined by measuring firefly luciferase by luminescence. Data was normalized to Renilla luciferase. Data correspond to the mean and SEM of 6 independent experiments. (**C**) Parental 16HBE and 16HBE *COPB1*KO cells were infected with SARS-CoV-2 HK (MOI = 1) in a synchronized manner. 6 h and 10 h post-infection, total RNA was extracted, and SARS-CoV-2 genome copy numbers were determined by RT-qPCR using primer pairs specific for *NSP12* and *NSP6*. SARS-CoV-2 RNA was expressed as the inverse of the raw Cq value for each replicate and time point. (**D**) Parental and *COPB1*KO HEK293T-ACE2-30F-PLP2 cells were infected with SARS-CoV-2 HK (MOIs 2 and 5). 4 h later, virus entry was examined by measuring firefly luciferase by luminescence. Data was normalized to Renilla luciferase. Data correspond to the mean and SEM of 4 independent experiments. **: $p < 0.01$; ****: $p < 0.0001$.

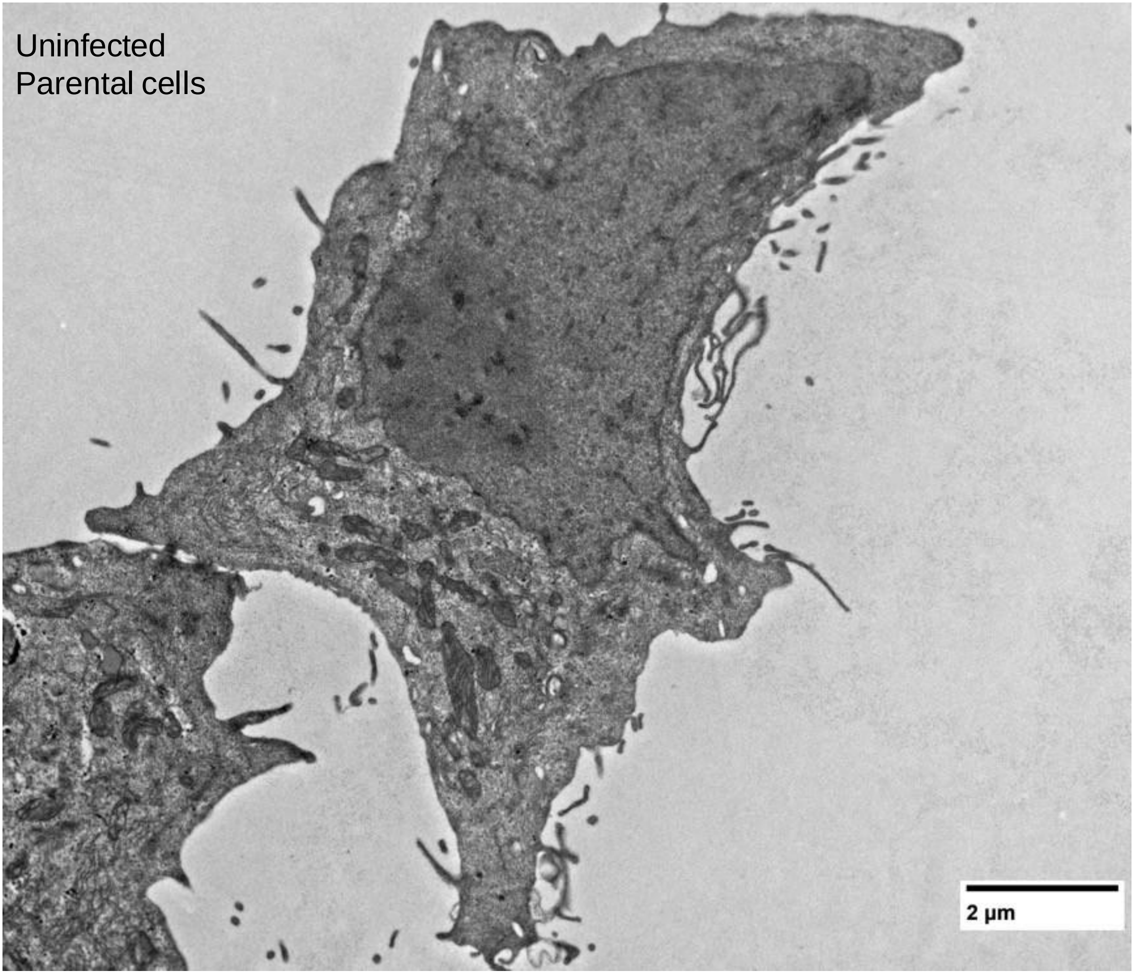
Uninfected
Parental cells
2 μm

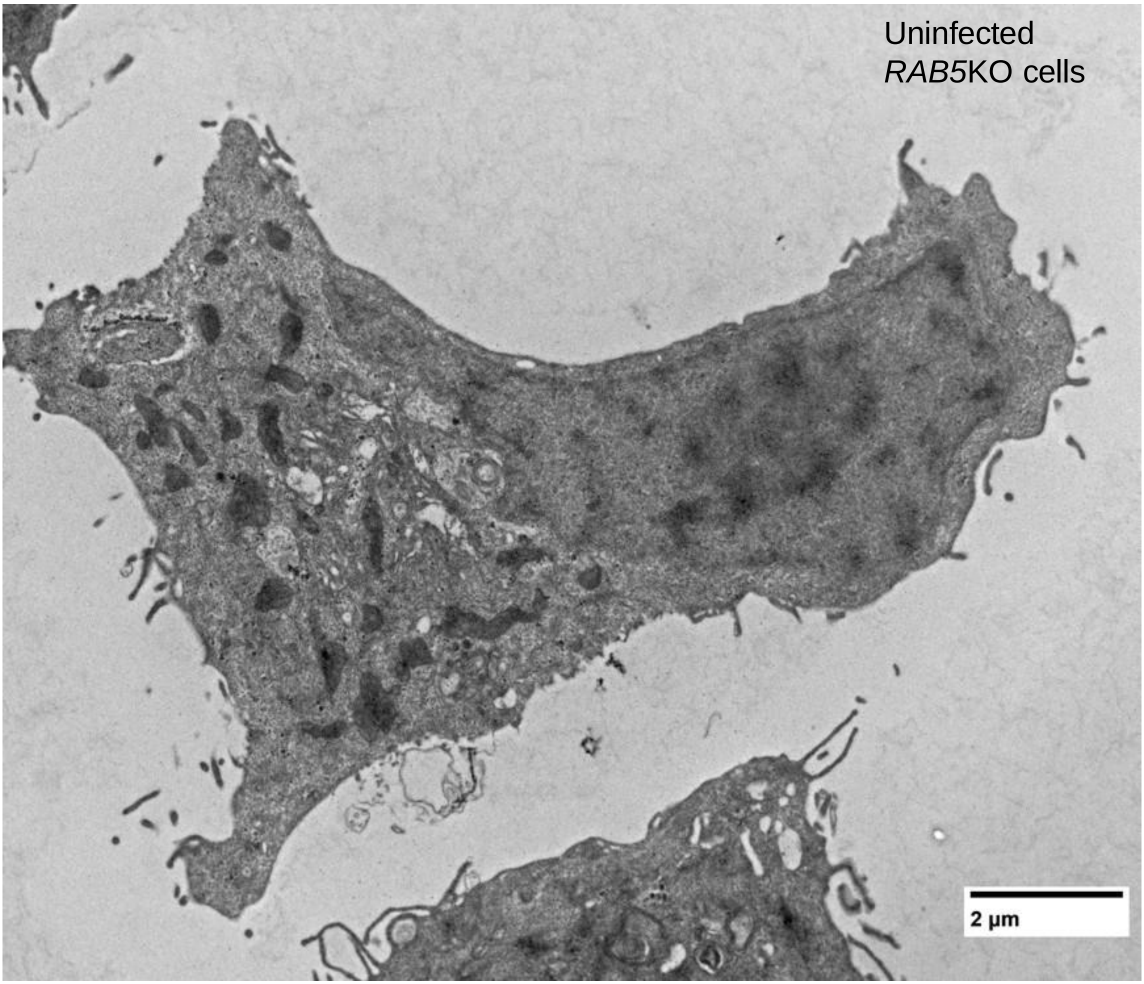
Uninfected
*RAB5*KO cells
2 μm

FIG S10. No evidence of juxtaposed DMVs nor maze-like structures in uninfected, parental and *RAB5*KO cells. To assess whether parental and *RAB*5KO VeroE6 cells can induce juxtaposed double membrane vesicles in the absence of SARS-CoV-2 infection, and whether the maze-like structures observed in infected, *RAB5*KO cells are an artifact due to the deletion of this gene, uninfected Parental and *RAB5*KO cells were analyzed by TEM. Top: Uninfected, Parental cells. Bottom: Uninfected, *RAB5*KO cells. Scale bar: 2 μm. Images are representative of 3 independent experiments.

REFERENCES

Borchers AC, Janz M, Schafer JH, Moeller A, Kummel D, Paululat A, Ungermann C, Langemeyer L. 2023. Regulatory sites in the Mon1-Ccz1 complex control Rab5 to Rab7 transition and endosome maturation. Proc Natl Acad Sci U S A 120:e2303750120.

Rink J, Ghigo E, Kalaidzidis Y, Zerial M. 2005. Rab conversion as a mechanism of progression from early to late endosomes. Cell 122:735-49.

Poteryaev D, Datta S, Ackema K, Zerial M, Spang A. 2010. Identification of the switch in early-to-late endosome transition. Cell 141:497-508.
